# RAB33A promotes metastasis via RhoC accumulation through non-canonical autophagy in cervical cancer

**DOI:** 10.1038/s41419-025-07455-w

**Published:** 2025-02-25

**Authors:** Lanqing Huo, Xiaodan Huang, Ying Wang, Yi Ouyang, Xueping Zheng, Yingyi Ouyang, Xinping Cao, Kai Chen, Denghui Wei, Yuanzhong Wu, Ruhua Zhang, Yujie Lin, Tiebang Kang, Ying Gao

**Affiliations:** 1 State Key Laboratory of Oncology in South China, Guangdong Provincial Clinical Research Center for Cancer, Sun Yat-sen University Cancer Center, Guangzhou, Guangdong PR China; 2 Department of Radiation Oncology, Sun Yat-sen University Cancer Center, Guangzhou, Guangdong PR China

**Keywords:** Macroautophagy, RHO signalling, Cervical cancer

## Abstract

Cervical cancer metastasis is characterized by the systemic spread of tumor cells. However, the underlying mechanism remains incompletely understood. Herein, we demonstrate that RAB33A promoted metastasis by enhancing RhoC accumulation and that higher RAB33A expression predicted poorer prognosis in patients with cervical cancer. Mechanistically, RhoC typically degraded via canonical autophagy due to the binding of two LIR motifs (LC3 interaction region) in RhoC to LC3; however, RAB33A induced non-canonical autophagy, resulting in RhoC stabilization, which facilitated pseudopodia formation and consequently cervical cancer metastasis. The fusion of RAB33A-induced autophagosomes with lysosomes was impaired, as RAB33A inactivated RAB7 by interacting with TBC1D2A, a GTPase-activating protein that targets RAB7. Our findings reveal a pivotal role of the RAB33A-RhoC axis in cervical cancer metastasis, indicating that RhoC inhibitors may be beneficial for treating cervical cancer patients with high levels of RAB33A.

## Introduction

Cervical cancer is one of the most common gynecological malignancies, with approximately 600,000 new cases and 340,000 cervical cancer-related deaths worldwide each year [1–3]. Although a treatment strategy based on concurrent chemoradiotherapy has improved local control and survival metrics, the prognosis of patients with cervical cancer remains poor; the 5-year mortality rate remains above 35%, and for patients with advanced-stage disease, this rate increases to nearly 50% [4, 5]. Notably, the primary obstacle to the effective management of cervical cancer is its distant metastasis, highlighting the urgent need for innovative strategies to overcome this challenge.

The Rho subfamily, which includes RhoA, RhoB, and RhoC, plays a pivotal role in tumor metastasis by cycling between their activated (GTP-bound) and inactivated (GDP-bound) forms to modulate cytoskeletal activities [6–8]. Similarly, members of the Rab GTPase family, which localize to distinct subcellular compartments, orchestrate specific vesicular transport pathways to contribute to cancer metastasis by regulating multiple cellular processes, such as cytoskeletal dynamics, cell motility, and extracellular matrix remodeling. For instance, Rab31 promotes metastasis in gastric adenocarcinoma [9]; moreover, Rab22a-NeoFs increases RhoA-GTP by binding to SmgGDS-607, which is a GTP-GDP exchange factor for RhoA, thereby promoting osteosarcoma metastasis [10]. On the other hand, members of the Rab GTPase family are also involved in the formation of autophagosomes, and this process is mainly regulated by the autophagy-related genes(ATG), especially ATG12, ATG5, and LC3-II complexes [11]. Under normal conditions, canonical autophagosomes generally fuse with lysosomes to degrade damaged organelles, protein aggregates, and intracellular pathogens [12]. Non-canonical autophagy (NCA) typically proceeds without the involvement of certain ATG genes but effectively recruits LC3 molecules to diverse vacuoles, as exemplified by LC3-associated phagocytosis (LAP) [13], LC3-associated endocytosis [14], and entosis [15]. Notably, xenophagy and LAP, which are distinct autophagic processes, have been demonstrated to phagocytose invading pathogens and subsequently degrade them through lysosomal fusion, and these processes occur in an LC3-dependent manner [13–16]. However, disruptions in this balance can result in the accumulation of non-degradative autophagic vacuoles [17], in which autophagosome–lysosome fusion is compromised or lysosomal degradation becomes inefficient. The resulting accumulation of damaged cellular factors potentially leads to disease onset and progression [18–20]. Autophagy can facilitate cancer cell resistance to factors such as nutrient scarcity and hypoxia, which are prevalent in the tumor milieu, but aberrant autophagy might induce tumor cell apoptosis and/or metastasis, providing potential therapeutic opportunities [4, 5, 21, 22]. The multifaceted role of autophagy in oncogenesis, especially in cervical cancer, may lead to the development of new treatment strategies.

In this report, we demonstrated that the binding of RhoC to LC3 occurs via its LIR motif, and this interaction leads to its degradation via the canonical autophagic lysosomal pathway normally. However, RAB33A inactivates RAB7, thereby inducing non-canonical autophagy to promote the accumulation of the RhoC protein, which in turn induces pseudopodia formation to promote cervical cancer metastasis.

## Materials and methods

### Cell culture

The human HeLa, SiHa, and HEK-293T cell lines were obtained from the American Type Culture Collection (ATCC). The cells were cultured in DMEM (Gibco) supplemented with 10% FBS (ExCell Bio) and 100 U/mL penicillin‒streptomycin at 37 °C in a 5% CO_2_ incubator. Notably, within six months before the project began, all the cell lines were authenticated by short tandem repeat profiling, and the cell lines were maintained in culture for no longer than one month.

### Antibodies

A comprehensive array of antibodies was used, and these antibodies and their sources were as follows:

Anti-ATG7 (ab133528, 1:2000) was obtained from Abcam. Anti-RhoC (34301; 1:1000), anti-β-tubulin (2128; 1:5000), anti-β-actin (4970; 1:5000), anti-V5-Tag (13202; 1:500), anti-Flag (14793; 1:2000), anti-Flag (8146; 1:2000), anti-HA (3724; 1:2000), anti-HA (2367; 1:2000), anti-ATG5 (12994; 1:2000), and anti-ATG16L1 (8089 T; 1:2000) were obtained from Cell Signaling Technology. Anti-RAB33A (GTX 55915; 1:500), anti-WIPI2 (GTX132453; 1:2000) and anti-TBC1D2A (GTX56123; 1:500) were obtained from GeneTex (GTX). Anti-RhoC (67542-1-Ig; 1:400), Anti-p62 (18420-1-AP; 1:2000), anti-GAPDH (10494-1-AP; 1:5000) and anti-rabbit IgG (30000-0-AP; 1:2000) were obtained from Proteintech Group (PTH). Anti-mouse IgG (sc-2025; 1:5000) was obtained from Santa Cruz Biotechnology. Anti-LC3B (L7543; 1:2000) was obtained from Sigma-Aldrich. The secondary antibodies conjugated to Alexa Fluor 594 (A-11037, A-11037; 1:500), Alexa Fluor 647 (A-21236; 1:500), and secondary antibodies 488 (A-11034, A32723; 1:500) were obtained from Invitrogen.

### Chemicals

CQ and 3-MA were obtained from Sigma-Aldrich (St. Louis, MO, USA). Baf A1 was obtained from LC Laboratories (Woburn, MA, USA). MG132 was obtained from Selleckchem (Houston, TX, USA).

### Plasmid construction

The GFP-RFP-LC3 plasmid was a generous gift from Dr. Min Li of Sun Yat-sen University [23]. RAB33A cDNAs were PCR-amplified and integrated into a pSIN vector with optional tags such as HA or Flag. For RNA targeting, the pLKO.1-puro vector was utilized, with sgRNA sequences informed by the GUIDES application. All the constructs were validated via Sanger sequencing. sgRNA sequences for CRISPR knockout and shRNA sequences for knockdown were shown in Supplementary Table 1.

### Transfection, lentivirus production, and stable cell line establishment

For the transient transfection experiments, plasmids were introduced into cells using either Lipofectamine 3000 (Life Technologies) or polyethyleneimine (PEI) (Polysciences). The medium was changed 6 h post transfection, and subsequent experiments were conducted 24–36 h later.

Lentivirus production for the gene overexpression, sgRNA, and shRNA experiments was performed as follows. Initially, HEK-293T cells were seeded and allowed to adhere for 24 h before cotransfection with 3 μg of lentiCRISPRv2-sgRNA/pSin-EF2-cDNA, 2 μg of psPAX2 (gag, pol), 1 μg of pMD2G, and 20 μL of PEI (2 mg/mL). After 48 h of incubation, the viruses were collected and subsequently filtered through 0.45-μm filters (Millipore). Virus-infected cells were generated by infecting cells in six-well plates with the appropriate viral titers in the presence of 10 μg/mL polybrene (Sigma) and centrifuging at 2000 rpm for 1 h at 37 °C. Finally, stable cell lines were selected by treatment with 0.5 μg/mL puromycin. Cells were transfected with siRNAs (Ribobio) using RNAiMAX (Thermo Fisher). Each 5 μL siRNA, 5 μL RNAimix and 200 μL Opti-MEM were mixed for each well of cells and the culture medium was refreshed after 6 h. The mRNA level of the target gene was quantified 72 h after transfection.

### Immunoblotting and coimmunoprecipitation (Co-IP)

For western blotting analysis, cells were first collected and subsequently lysed on ice in RIPA buffer (150 mM NaCl, 0.5% EDTA, 50 mM Tris-HCl, 0.5% NP40). The lysis buffer was supplemented with Protease Inhibitor Cocktail Set I (Calbiochem; 539131) and Phosphatase Inhibitor Cocktail Set II (Calbiochem; 524625). The lysates were then centrifuged at 12,000 rpm for 15 min at 4 °C, after which the protein concentrations were measured by Bradford assay. The protein samples were loaded and separated on 12% sodium dodecyl sulfate-polyacrylamide gels, and then, the proteins were transferred onto PVDF membranes (Millipore). The membranes were blocked in PBS with 5% nonfat milk and 0.1% Tween-20 and probed with primary antibodies overnight at 4 °C, followed by incubation with secondary HRP-conjugated antibodies for 1 h at room temperature. The protein bands were visualized using High-sig ECL substrate (Tanon) and imaged using a MiniChemi chemiluminescence imager (SAGECREATION, Beijing). These results are presented from three independent experiments.

For Co-IP experiments, the supernatants were initially incubated with washed anti-Flag agarose beads (Selleck, B23102) for 1.5 h at 4 °C, followed by six washes with RIPA buffer prior to western blotting analysis. These results are presented from three independent experiments.

### Immunofluorescence

For immunofluorescence staining, cells were fixed with 4% paraformaldehyde for 15 min, permeabilized with 0.5% Triton X-100 (Sigma-Aldrich) for 15 min, and blocked with goat serum (ZSGB-BIO, ZLI-9056) for 30 min at room temperature. After blocking, the cells were incubated with primary antibodies for 2 h at room temperature or overnight at 4 °C, followed by three washes with PBS. Subsequently, the cells were stained with secondary antibodies for 1 h and with Hoechst 33342 (Invitrogen, H3570) for 2 min. After staining, the cells were washed three times with PBS and mounted with antifade mounting medium (Beyotime, P0128M). Images were acquired using a confocal microscope (Nikon CSU-W1) with a 100× oil immersion objective lens (1.49 NA; Nikon). Image acquisition and reconstruction were performed using Nikon NIS-Elements. We employed the intensity profile method to assess colocalization between fluorescent channels. Fluorescence intensity was measured along linear or segmented lines across regions of interest within cells, typically covering areas containing multiple channels. By examining the overlap and correlation of intensity peaks for distinct channels along these profiles, we inferred the degree of spatial colocalization between proteins. Data were analyzed using GraphPad Prism 9.

### qPCR

Total RNA was extracted using an RNA extraction kit (TIANGEN), and cDNA was synthesized according to the manufacturer’s instructions (Tarkara). qRT-PCR was performed using a Light Cycler 480 instrument (Roche Diagnostics) with SYBR Green PCR Master Mix (Kapa). All reactions were carried out in a 10 µL reaction volume in triplicate. The primers for glyceraldehyde 3-phosphate dehydrogenase (GAPDH) were obtained from Invitrogen. Standard curves were generated, and the relative amount of target gene mRNA was normalized to that of GAPDH. The specificity was verified by melting curve analysis. The primers were used as below:

Human RhoC: 5’-GGAGGTCTACGTCCCTACTGT-3’ and 5’- CGCAGTCGATCATAGTCTTCC-3’;

GAPDH: 5’-GGAGCGAGATCCCTCCAAAAT-3’ and 5’-GGCTGTTGTCATACTTCTCATGG-3’

### RNA-seq

RNA-seq of 6 cervical cancer tissues was performed by Novogene using Illumina X TEN. The 6 GB clean data per sample was collected for RNA-seq. Hg38 assembly was used for the read alignment, and gene annotation was obtained using Ensembl gene annotation version 90. The 6 GB clean data per sample was collected for RNA-seq, and the clean reads were aligned to the human genome GRCh38 (Hg38) using hisat2 (version 2.0.5).

### Structural variants/mutants

We generated structural variants of TBC1D2A by constructing truncated forms of its functional domains. Specifically, the PH truncation connected the amino acid at position 45 directly to that at position 142, effectively removing the intervening sequence. The CC truncation linked the amino acid at position 298 to the amino acid at position 416, while the TBC truncation connected the amino acid at position 625 to that at position 817. For the expression of these constructs, we cloned the truncated domains into the pSin expression vector, ensuring that each domain was properly positioned within the vector framework. The cloning process involved PCR amplification of the desired fragments, followed by ligation into the pSin vector with optional V5 tag.

We generated structural variants of RhoC by mutating specific sequences within its structure. The FPEV motif (amino acids 30–33) was substituted with APEA, the YDRL motif (amino acids 66–69) with ADRA, and the FGYL motif (amino acids 154–157) with AGYA, resulting in the constructs RhoC-LIR-1, RhoC-LIR-2, and RhoC-LIR-3, respectively. Additionally, we introduced alanine mutations at positions 66, 69, 154, and 157 to create the RhoC-LIR-2 + 3 variant.

### Migration and invasion assays

The 24-well Boyden chambers with 8-μm inserts coated with Matrigel for invasion or uncoated for migration. A total of 1 × 10^5^ cells per well were plated on the inserts and cultured at 37 °C in the upper chambers without fetal calf serum. The upper chambers lie in the 24-well Boyden chambers with DMEM containing 15% fetal calf serum. After 14 h, cell inserts were fixed with 4% PFA for 10 min, followed by PBS wash and crystal violet (0.005%, Sigma) staining to allow visualization and counting. Calculate the number of stained cells in each field of view for statistical analysis under phase-contrast microscopy. The data were presented as the mean ± SD. The error bars indicated the SD.

### Animal experiments

Animal care and experiments followed the “Guide for the Care and Use of Laboratory Animals” and the “Principles for the Utilization and Care of Vertebrate Animals.” All the procedures were approved by the Animal Research Committee of Sun Yat-sen University Cancer Center (L102042022060F). Female BALB/c nude mice, aged 4 weeks, were obtained from Beijing Wei Tong Li Hua Lab Animal Technology Co., Ltd. In the lymph node metastasis model, 5 × 10^5^ SiHa cells were injected into the footpads of BALB/c nude mice. After 6 weeks, the mice were euthanized, and the popliteal and inguinal lymph nodes were harvested for analysis. All the dissected tissue samples were paraffin-embedded, sectioned, and stained with H&E. We calculated lymph node volumes by measuring the length (*L*, the longer dimension), width (W, the shorter dimension, perpendicular to the plane of length and parallel to the animal’s body plane), and height (H, the distance between the upper boundary of the lymph node and the animal’s body) using a caliper. The volume of each individual lymph node was calculated using the formula: Volume = 1/6 × π × L × W × H.

### Human tissue specimens

For expression analysis, 6 freshly frozen cervical cancer samples were obtained from Sun Yat-sen University Cancer Center (SYSUCC). For prognosis analysis, another 50 cervical cancer biopsy tissue samples were collected from patients with detailed clinical characteristic information and long-term follow-up data at SYSUCC from 2015 to 2018. None of the patients received any antitumor treatment prior to biopsy. The use of human cervical carcinoma tissues was reviewed and approved by the ethical committee of SYSUCC (Approval No. B2023-041-01) in accordance with the Declaration of Helsinki, and informed consent was obtained from the patients. The samples were retrospectively acquired from the Department of Radiation Oncology, archives of SYSUCC.

### Immunohistochemistry

Freshly collected tumor samples were fixed in 4% paraformaldehyde and subsequently embedded in paraffin before being sectioned into 4-μm-thick sections. The sections were deparaffinized, subjected to antigen retrieval, and blocked. The primary antibodies against RAB33A (GTX, 55915) were diluted 1:100 and against RhoC (PTH, 67542) were diluted 1:400. For immunohistochemical analysis, the sections were incubated with anti-RAB33A and anti-RhoC antibodies for 2 h at room temperature, followed by incubation with anti-rabbit IgG secondary antibodies and DAB reagent. Immunohistochemical (IHC) staining was assessed and scored separately by two independent investigators blinded to the clinic pathological data. Protein expression levels of RAB33A were evaluated using a 13-point scoring system. A semiquantitative scoring criterion was applied to quantify RAB33A and RhoC expression, incorporating both staining intensity and the extent of positive areas. The staining index (range: 0–12) was calculated by multiplying the intensity of positive staining (weak, 1; moderate, 2; strong, 3) with the percentage of immunopositive cells in the target area (0%, 0; <10%, 1; 10–50%, 2; 51–80%, 3; >80%, 4). The cutoff values for high or low expression levels of the indicated molecule were established based on receiver operating characteristic (ROC) curve analysis.

### Statistical analysis

Survival data and patient correlations were statistically analyzed using Kaplan–Meier plots, the log-rank test, and Cox regression models. Experimental data analysis was conducted with the SPSS software package, and statistical significance was defined as *P* < 0.05. Mean values for the control and experimental groups were compared to assess significant differences, with data presented as the mean ± SD, and error bars representing SD. The statistical significance of group differences was evaluated using an unpaired, two-tailed Student’s *t*-test and two-way analysis of variance (ANOVA).

## Results

### RAB33A promotes cervical cancer metastasis

**Fig. 1:**
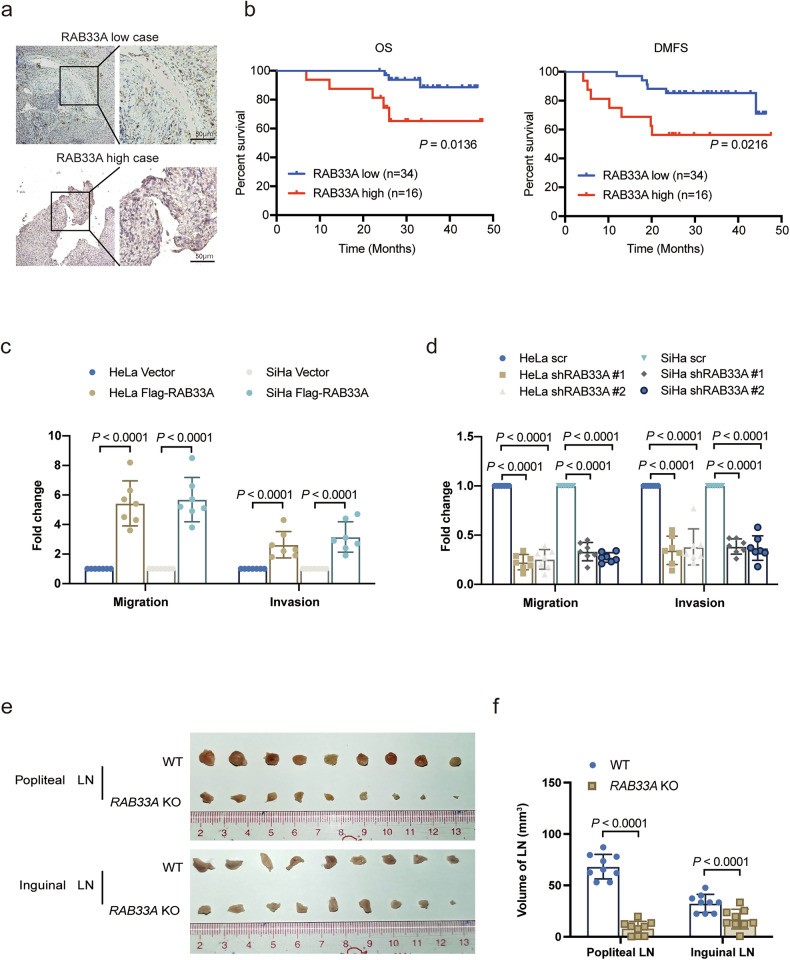
RAB33A enhances metastatic potential in cervical cancer. **a** Immunohistochemical staining for RAB33A in cervical carcinoma specimens delineating cases without metastasis or recurrence from those with distant metastasis. **b** Overall survival (OS) and distant metastasis-free survival (DMFS) curves based on RAB33A protein levels in 50 paraffin-embedded cervical carcinoma tissues; patients with elevated RAB33A expression had reduced overall survival (*P* = 0.0136) and an increased risk of distant metastasis (*P* = 0.0216). **c** Migration and invasion of HeLa or SiHa cells overexpressing RAB33A. **d** Migration and invasion of HeLa or SiHa cells with or without RAB33A knockdown. **e**, **f** In vivo model of cervical cancer lymph node metastasis established using the indicated stable cell lines; the model was established in 9 biologically independent mice. Dissected popliteal and inguinal LNs (**e**) and their volumes (**f**) from mice with tumors derived from wild-type or *RAB33A*-knockout HeLa cells. The data are presented as the mean ± SD. *P* values are shown; two-tailed Student’s *t*-test. Vector vector-only control. WT wild type.

To determine the key molecules that are related to cervical cancer metastasis, RNA-Seq was performed on six tumor tissues from patients with metastasis and patients without metastasis. Among the genes whose expression levels were higher in metastatic patients, RAB33A caught our attention, as its role in metastasis has not been explored (Supplementary Fig. S1a). Moreover, cervical cancer patients with higher RAB33A expression according to IHC staining had shorter overall survival (OS) and distant metastasis-free survival (DMFS) (Fig. 1a, b).

Overexpression or knockout of RAB33A was performed in HeLa and SiHa cells, two cervical cancer cell lines (Supplementary Fig. S1b, d). Both cell migration and invasion, but not cell viability, were enhanced or reduced by the overexpression or knockdown of RAB33A, respectively (Fig. 1c, d, Supplementary Fig. S1c, e). Using a lymph node metastasis model in 4-week-old female nude mice (Supplementary Fig. S1f), we found that cervical cancer cells overexpressing RAB33A resulted in larger popliteal and inguinal metastatic lymph nodes, whereas *RAB33A*-knockout cervical cancer cells resulted in smaller popliteal and inguinal metastatic lymph nodes (Fig. 1e, f, Supplementary Fig. S1g, h). Taken together, our results indicated the pivotal role of RAB33A in promoting cervical cancer metastasis.

### RAB33A promotes migration and invasion by increasing the levels of active RhoC

**Fig. 2:**
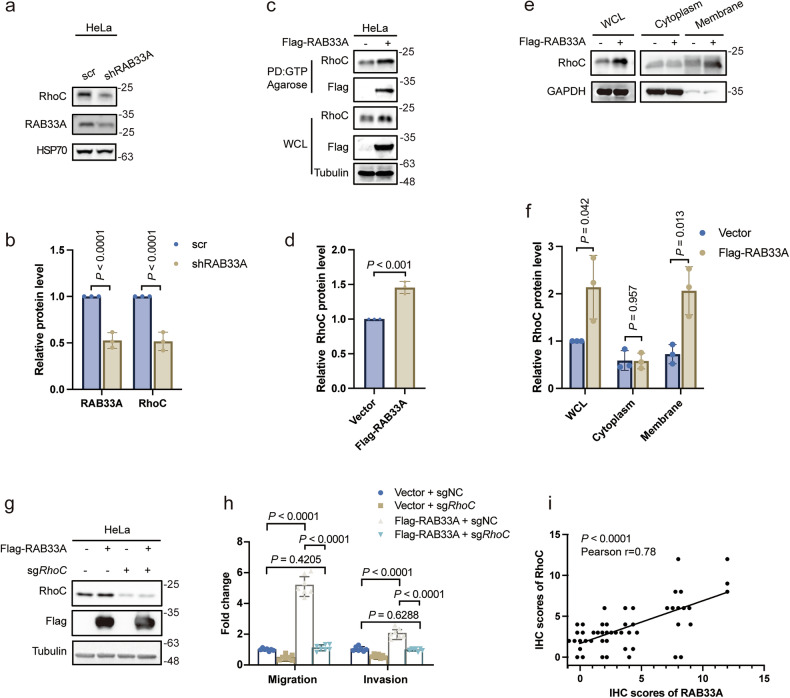
RAB33A-mediated cervical cancer metastasis is dependent on RhoC. **a**, **b** Western blotting analysis of the RhoC protein levels in RAB33A-knockdown HeLa cells. These results are presented from three independent experiments. The protein levels of RAB33A/RhoC were then quantified (mean ± SD. n = 3 times. two-tailed Student’s *t*-test). **c**, **d** A GTP pull-down assay revealed increased levels of active RhoC in RAB33A-overexpressing cells. Active RhoC in wild-type or RAB33A-overexpressing HeLa cells was detected by a GTP pull-down assay. These results are presented from three independent experiments. The protein levels of RhoC in the pull-down assays were then quantified (mean ± SD. n = 3 times. Two-tailed Student’s *t*-test). **e**, **f** Elevated RhoC expression in the cytoplasm and organelle membranes was observed via western blotting. The cytoplasm and membrane fractions from wild-type or RAB33A-overexpressing HeLa cells were separated. The RhoC protein levels were determined by western blotting. These results are presented from three independent experiments. The protein levels of RhoC were then quantified (mean ± SD. n = 3 times. two-tailed Student’s *t*-test). **g** Western blotting analysis of knockout *RhoC* in wild-type or RAB33A-overexpressing HeLa cells. **h** Migration and invasion assays were performed using the indicated stable cell lines. These results are presented from three independent experiments. **i** The correlation of the IHC scores of RAB33A and RhoC, *n* = 50 patients.

In fact, the morphology of RAB33A-overexpressing cervical cancer cells was altered, and RAB33A increased the formation of both linear and lamellar pseudopods according to phalloidin staining results (Supplementary Fig. S2a); these results indicated that Rho family members, which are involved in cytoskeletal modulation, may participate in the promotion of migration and invasion by RAB33A in cervical cancer cells. Indeed, RhoC was significantly increased, while RhoA and RhoB were modestly elevated, by RAB33A overexpression in HeLa cells (Supplementary Fig. S2b). Consistent with these findings, RAB33A knockdown decreased RhoC levels in HeLa cells (Fig. 2a, b). Moreover, by performing a GTP pull-down assay, we found that the levels of RhoC-GTP were increased by RAB33A overexpression, indicating that RhoC is more active in cells overexpressing RAB33A (Fig. 2c, d). This was further confirmed by the increase in the membrane localization of RhoC in cells overexpressing RAB33A (Fig. 2e, f, Supplementary Fig. S2c), as RhoC is translocated into the membrane when it is activated [24]. However, the RAB33A overexpression-induced increases in both migration and invasion were abolished in *RhoC*-knockout HeLa cells (Fig. 2g, h). Positive correlation of IHC score between RAB33A and RhoC could be observed (Fig. 2i, Supplementary Fig. S2d). Collectively, these results demonstrated that RAB33A promoted migration and invasion by increasing the levels of active RhoC in cervical cancer cells.

### RhoC is degraded by canonical autophagy via the binding of two LIR motifs in RhoC to LC3

**Fig. 3:**
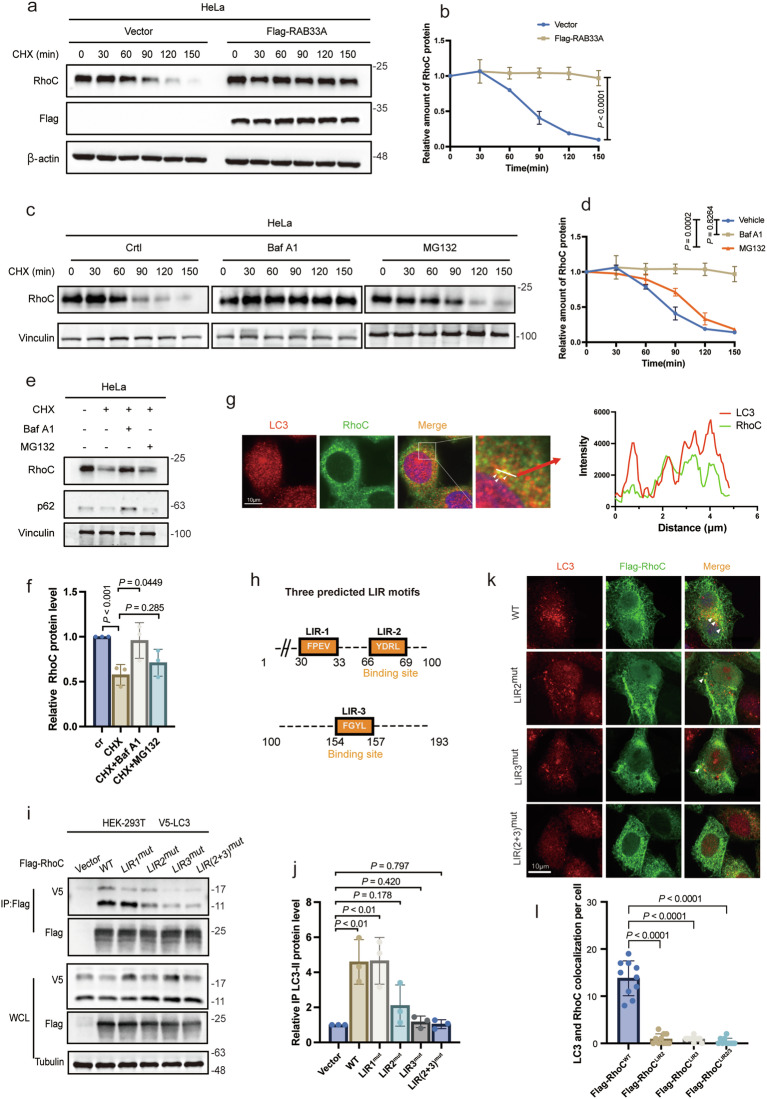
RhoC interacts with LC3 via LIR motifs and undergoes autolysosomal degradation. **a** Western blotting analysis of the RhoC protein levels in wild-type or RAB33A-overexpressing HeLa cells treated with 40 μg/mL CHX. These results are presented from three independent experiments. **b** Relative quantitation of RhoC protein levels was based on the western blotting results. (*P* < 0.0001). **c** Western blotting analysis of RhoC protein levels in HeLa cells treated with 40 μg/mL CHX, 100 nM Baf A1 and 10 μM MG132. These results are presented from three independent experiments. **d** Relative quantitation of RhoC protein levels was based on the western blotting results. (*P* < 0.0001, *P* = 0.8264). **e**, **f** RhoC undergoes autophagy-mediated degradation (CHX: 40 μg/mL, 6 h; Baf A1: 100 nM, 6 h; MG132: 10 μM, 6 h). HeLa cells were treated with the indicated inhibitors for 6 h, after which the RhoC protein levels were determined via western blotting. These results are presented from three independent experiments. The protein levels of RhoC were then quantified (mean ± SD. *n* = 3 times. two-tailed Student’s *t-*test). **g** LC3 and RhoC colocalization was visualized by immunofluorescence. Arrowheads indicate the colocalization of the two proteins. **h** Three predicted LIR sequences in RhoC. **i**, **j** Flag immunoprecipitates (IP) from lysates of HEK293T cells expressing V5-LC3 and Flag-tagged RhoC LIR motif mutants. These results are presented from three independent experiments. The protein levels of IP LC3-II were then quantified (mean ± SD. *n* = 3 times. two-tailed Student’s *t*-test). **k**, **l** Immunofluorescence analysis of the colocalization of LC3 and RhoC proteins with mutated LIR motifs. The colocalization of the LC3 and RhoC proteins with mutated LIR motifs was quantified (*n* = 10 cells). The data are presented as the mean ± SD. *P* values are shown; Student’s *t*-test.

Next, we sought to investigate how RAB33A increases RhoC levels in cervical cancer cells. Interestingly, RAB33A overexpression had no effect on the mRNA level of RhoC (Supplementary Fig. S3a), suggesting that RAB33A may regulate RhoC at the posttranslational level. We did observe that RhoC was an unstable protein, as its half-life was 90 min (Supplementary Fig. S3b), and that RhoC was stabilized by RAB33A overexpression in both HeLa and SiHa cells (Fig. 3a, b, Supplementary Fig. S3c). The degradation of RhoC was prevented by treatment with the lysosome inhibitor bafilomycin A1 (Baf A1) but not by treatment with the proteasome inhibitor carbobenzoxy-Leu-Leu-leucinal (MG132) (Fig. 3c–f). These results demonstrated that RAB33A increases the protein level of RhoC by preventing its degradation by lysosomes.

Since the RhoC protein is degraded by lysosomes, we hypothesized that autophagy may be involved in RhoC degradation. Colocalization of endogenous RhoC and LC3 was observed (Fig. 3g). There are three predicted typical LC3 interaction region (LIR) motifs (W/F/YxxL/I/V) in the protein sequence of RhoC [25], namely, FPEV (amino acids 30-33, LIR-1), YDRL (amino acids 66-69, LIR-2) and FGYL (amino acids 154-157, LIR-3) (Fig. 3h), and these LIR motifs were individually mutated. As shown in Fig. 3i–l, both the interaction and colocalization of RhoC with LC3 were reduced when the LIR-2 YDRL and LIR-3 FGYL mutants were expressed but not when the LIR-1 FPEV mutant was expressed. These results indicated that RhoC was degraded by canonical autophagy via the binding of two LIR motifs (LIR-2 and LIR-3) in RhoC to LC3.

### RAB33A induces non-canonical autophagy to stabilize RhoC, promoting cervical cancer metastasis

**Fig. 4:**
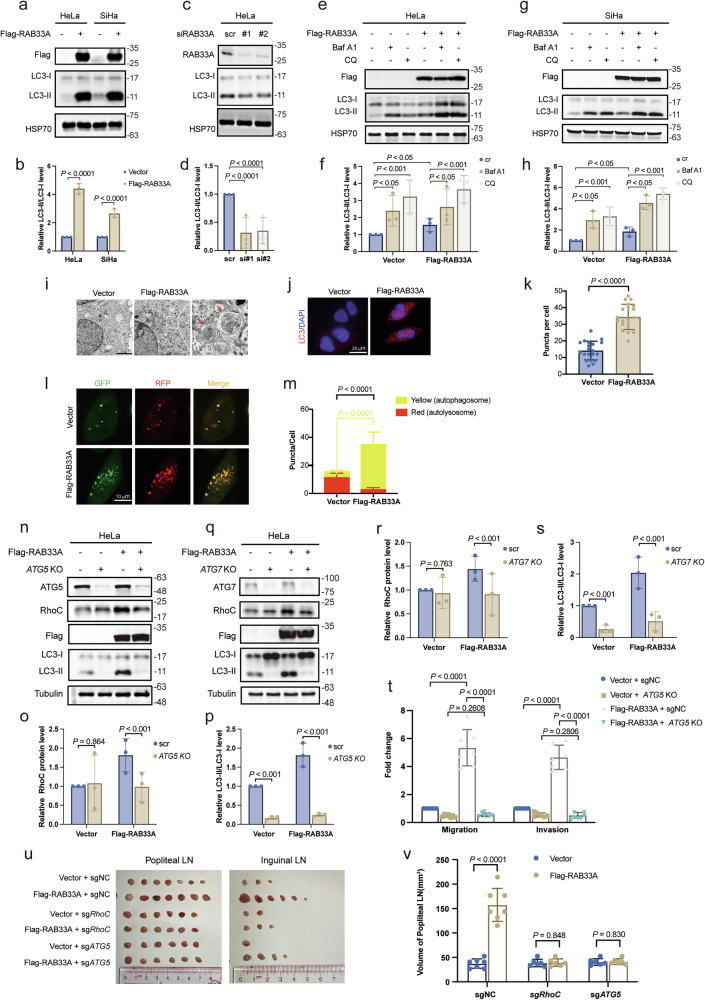
RAB33A induces nondegradative autophagy to stabilize RhoC. **a**–**d** LC3-II protein levels in wild-type or RAB33A-overexpressing HeLa and SiHa cell lines (**a**) and wild-type or RAB33A-knockdown HeLa and SiHa cell lines (**c**) were measured by western blotting. These results are presented from three independent experiments. The protein levels of LC3-II/LC3-I were then quantified (mean ± SD. n = 3 times. two-tailed Student’s *t*-test). **e**–**h** Increased LC3-II expression in cells treated with Baf A1 combined with CQ (Baf A1: 200 nM, 6 h; CQ: 40 μM, 6 h). Wild-type or RAB33A-overexpressing HeLa (**e**) and SiHa (**g**) cell lines were treated with the late autophagosome inhibitor Baf A1 (200 nM) or CQ (40 μM) for 6 h. The protein levels of LC3-II were determined by western blotting. These results are presented from three independent experiments. The protein levels of LC3-II/LC3-I were then quantified (mean ± SD. *n* = 3 times. two-tailed Student’s *t*-test). **i** Electron microscopic images; autophagosomes are indicated by red arrows. **j**, **k** Immunofluorescence image of increased numbers of autophagosomes after RAB33A overexpression (*P* < 0.0001). Localization of endogenous LC3 in HeLa cells (**j**), and the number of LC3 puncta per cell was calculated (**k**, *n* = 20 cells). The data are presented as the mean ± SD. *P* values are shown; two-tailed Student’s *t*-test. Vector, vector-only control. **l**, **m** The colocalization of GFP and RFP puncta in live cells was quantified. The numbers of puncta, either yellow puncta with both green and red fluorescence (autolysosome) or red puncta with only red fluorescence (autophagosome), were quantified (right panel). *P* values were calculated by Student’s *t*-test. **n**–**s** Effects of *ATG5* (**n**) and *ATG7* (**q**) knockout combined with RAB33A overexpression on RhoC and LC3-II levels and migratory capabilities. RhoC and LC3-II protein levels in HeLa cells with *ATG5* or *ATG7* knockout combined with RAB33A overexpression. These results are presented from three independent experiments. The protein levels of RhoC and LC3-II/LC3-I were then quantified (mean ± SD. n = 3 times. two-tailed Student’s *t*-test). **t** Migration and invasion of HeLa cells with *ATG5* knockout combined with RAB33A overexpression. **u**, **v** An in vivo model of cervical cancer lymph node metastasis was established using the indicated stable cell lines; the model was established in 7 biologically independent mice. Dissected popliteal and inguinal LNs (**u**) and their volumes (**v**) from mice with tumors derived from wild-type or *RhoC/ATG5-*knockout SiHa cells. The data are presented as the mean ± SD. *P* values are shown; Student’s *t*-test.

Subsequently, we aimed to explore whether RAB33A is involved in autophagy. Notably, overexpression and knockdown of RAB33A resulted in an increase and decrease, respectively, in the expression of LC3-II, a marker of autophagy (Fig. 4a–d). The induction of LC3-II by RAB33A overexpression was enhanced by treatment with either Baf A1 or chloroquine (CQ) (Fig. 4e–h); these results indicated that RAB33A may induce autophagy. The induction of autophagy by RAB33A overexpression was confirmed by electron microscopy and confocal microscopy (Fig. 4i–k). However, the autophagy induced by RAB33A overexpression in cervical cancer cells was not inhibited by 3-methyladenine (3-MA), a PI3K inhibitor, (Supplementary Fig. S4a, b) and was independent of both Beclin1 (BECN1) and WD repeat domain, phosphoinositide interacting 2 (WIPI2) (Supplementary Fig. S4c, d). Moreover, the colocalization of GFP and RFP puncta showed that autophagosomes accumulated when RAB33A was overexpressed in cervical cancer cells (Fig. 4l, m). These results revealed that RAB33A-induced autophagy in cervical cancer cells was a type of non-canonical autophagy.

Consistently, knockout of *ATG5*, *ATG7* or *ATG16L1*, which are the genes required for the formation of autophagosomes [26], abrogated the RAB33A-induced increase in both RhoC and LC3-II levels (Fig. 4n–s, Supplementary. S4e). Notably, the promotion of migration and invasion by RAB33A overexpression was reversed by knocking out *ATG5* in cervical cancer cells (Fig. 4t). Using the lymph node metastasis model as mentioned above, we found that cervical cancer cells overexpressing RAB33A resulted in larger popliteal metastatic lymph nodes, while knocking out *ATG5* or *RhoC* could reverse it (Fig. 4u, v). Collectively, our results showed that RAB33A induced non-canonical autophagy to stabilize RhoC, which in turn promoted metastasis in cervical cancer.

### RAB33A recruits TBC1D2A to inactivate RAB7 to inhibit RhoC degradation

**Fig. 5:**
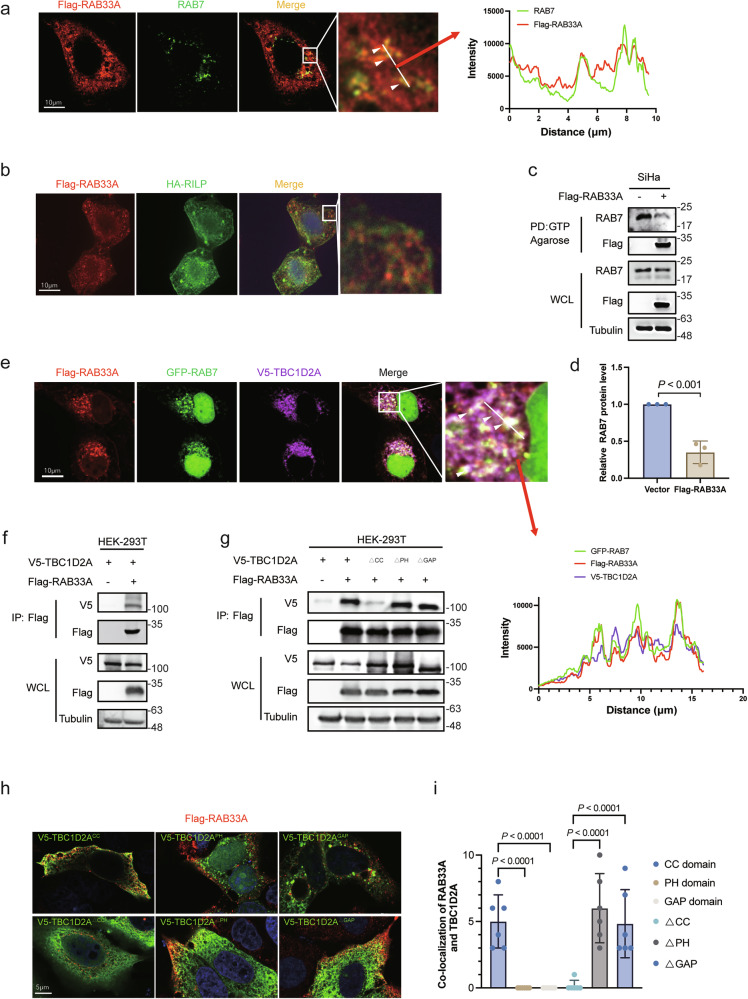
RAB33A-mediated recruitment of TBC1D2A inhibits RAB7 activation to inhibit RhoC degradation. **a** Localization of RAB33A and endogenous RAB7A in HeLa cells stably overexpressing RAB33A. Arrowheads indicate the colocalization of the two proteins. **b** Localization of RAB33A and HA-RILP in HeLa cells stably overexpressing RAB33A and transiently transfected with HA-RILP for 48 h. **c**, **d** Overexpression of RAB33A in SiHa cells results in reduced RAB7-GTP levels. Active RAB7 in wild-type or RAB33A-overexpressing SiHa cells was detected by a GTP pull-down assay. These results are presented from three independent experiments. The protein levels of RAB7 in the pull-down assay were then quantified (mean ± SD. *n* = 3 times. two-tailed Student’s *t*-test). **e** Localization of Flag-RAB33A, GFP-RAB7 and V5-TBC1D2A in HeLa cells stably overexpressing Flag-RAB33A and transiently transfected with GFP-RAB7 and V5-TBC1D2A for 48 h. The HEK-293T cell line was transiently cotransfected with V5-TBC1D2A (**f**), distinct TBC1D2A domains (**g**) and Flag-RAB33A for 48 h, after which the TBC1D2A-RAB33A interaction was assessed by western blotting. **h**, **i** The specific colocalization of RAB33A with distinct TBC1D2A domains. Localization of V5-tagged TBC1D2A mutants in HeLa cells stably expressing Flag-RAB33A (**h**). Cells were transiently transfected with the indicated plasmids for 48 h. The number of puncta indicating Flag-RAB33A and TBC1D2A colocalization in each cell was calculated (**i**, *n* = 6 fields). The data are presented as the mean ± SD. *P* values are shown; two-tailed Student’s *t*-test. Vector, vector-only control.

Given that RAB31 and RAB22A can inactivate RAB7, thereby preventing the fusion of multivesicular vesicles and autophagosomes with lysosomes, respectively [27, 28], we hypothesized that RAB33A, which is closely related to both RAB31 and RAB22A in the RAB family, may also inactivate RAB7. As shown in Fig. 5a, overexpressed RAB33A was colocalized with endogenous RAB7. However, overexpressed RAB interacting lysosomal protein (RILP), which is an effector protein that binds only to activated RAB7-GTP, did not colocalize with overexpressed RAB33A (Fig. 5b). Furthermore, the activity of RAB7 was reduced by RAB33A overexpression in HeLa and SiHa cells through GTP pull-down experiment (Fig. 5c, d, Supplementary Fig. S5a). These results demonstrated that RAB33A inactivated RAB7.

There are four GAPs that target RAB7, namely, TBC1D2A, TBC1D2B, TBC1D5, and TBC1D15 [28, 29]; among them, only TBC1D2A was shown to colocalize with RAB33A (Supplementary Fig. S5b, c), and triple colocalization of RAB33A, TBC1D2A and RAB7 was observed in HeLa cells **(**Fig. 5e). Meanwhile, the interaction of RAB33A with TBC1D2A was detected at their ectopic levels (Fig. 5f). TBC1D2A contains a pleckstrin homology (PH), a coiled-coil domain (CC) and GAP structural domains. We generated constructs that included only one domain each, named TBC1D2A^PH^, TBC1D2A^CC^ and TBC1D2A^GAP^, or in which each domain was deleted, called TBC1D2A^∆PH^, TBC1D2A^∆CC^ and TBC1D2A^∆GAP^. As shown in Fig. 5g, there is no interaction of RAB33A with TBC1D2A^∆CC^. Additionally, TBC1D2A^CC^, but not TBC1D2A^PH^ or TBC1D2A^GAP^, colocalized with RAB33A, while only TBC1D2A^∆CC^ did not colocalize with RAB33A (Fig. 5h, i); these results strongly suggested that RAB33A recruited TBC1D2A through its CC structural domain to inactivate RAB7 in cervical cancer cells.

## Discussion

**Fig. 6:**
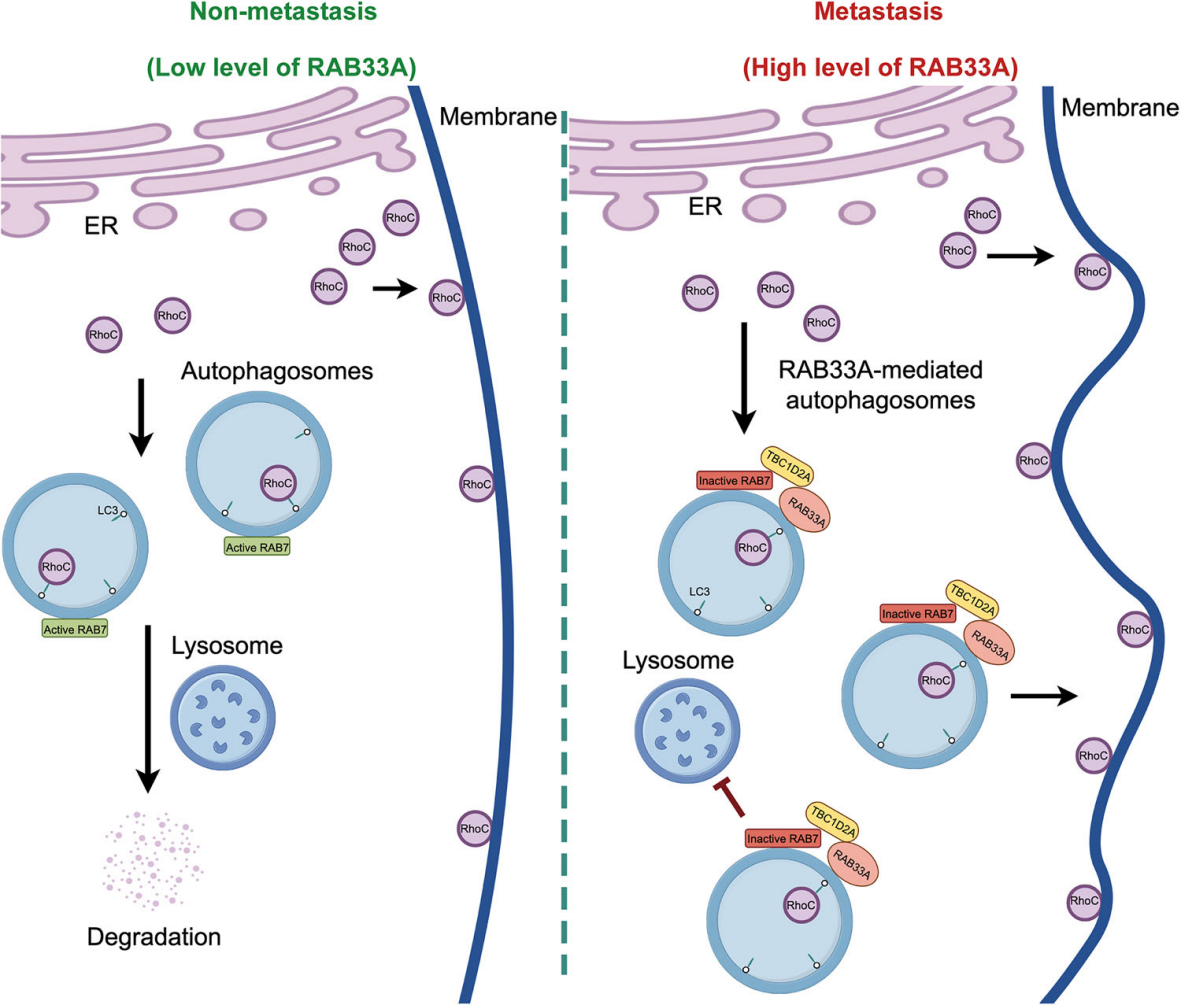
The proposed model for the RAB33A-RhoC axis in the metastasis of cervical cancer. In cervical cancer cells, RAB33A induces the formation of non-canonical autophagosomes and inhibits the fusion of autophagosomes and lysosomes by inactivating RAB7. This enables the accumulation of RhoC, which is degraded through the autophagy-lysosome pathway within the cell. The excess RhoC is transported to the cell membrane, altering cell morphology and promoting metastasis.

Cervical cancer metastasis remains a formidable challenge. As shown in Fig. 6, RhoC is degraded via canonical autophagy in nonmetastatic cervical cancer cells, and this process depends on two LIR motifs in RhoC. Conversely, in highly metastatic cervical cancer cells, RAB33A is overexpressed and induces non-canonical autophagy, recruiting TBC1D2A to inactivate RAB7, thereby inhibiting the fusion of RAB33A-induced autophagosomes with lysosomes. Consequently, in this case, RhoC accumulates through a noncanonical autophagy pathway, resulting in an increase in the formation of both linear and lamellar pseudopods, which in turn enhances cell migration, invasion, and metastasis. Our findings reveal the crucial role of the RAB33A-RhoC axis in cervical cancer metastasis and indicate that Rho inhibitors, such as fasudil hydrochloride, may benefit a subset of cervical cancer patients.

Given that Rho family members play crucial roles in metastasis, regulating the stability of these proteins is of paramount importance. For instance, the endolysosomal catabolism of RhoB depends on its C-terminal configuration and lipidic alterations [30], whereas RhoA undergoes ubiquitin‒proteasome degradation, which is regulated by the SCF (FBXL19) E3 ligase and Erk2-mediated phosphorylation [8, 31]. In this report, we showed that RhoC is degraded by an autophagic pathway via its interaction with LC3 through two LIR motifs. Interestingly, the LIR motif is also present in both the RhoA and RhoB proteins, and their levels were modestly increased by RAB33A overexpression in cervical cancer cells. These findings suggest that RhoA and RhoB may similarly undergo degradation via autophagy.

The formation of autophagosomes occurs through both canonical and non-canonical pathways. Canonical autophagy involves the participation of all ATGs, whereas non-canonical autophagy does not require the hierarchical involvement of ATGs, particularly BECN1 [32]. The primary phospholipid component in canonical autophagosomes is PI3P [33], whereas the phospholipid composition of non-canonical autophagosomes varies. For example, in RAB22A-induced non-canonical autophagosomes, the predominant phospholipid is PI4P, and such autophagosomes fuse with early endosomes to form Rafeesomes, whose inner vesicles become extracellular vesicles as R-EV [27, 33]. RAB33A-induced non-canonical autophagosomes, whose specific phospholipid components are being explored in our laboratory, do not fuse with lysosomes, and we hypothesize that these autophagosomes may fuse with the cell membrane, as the amount of active RhoC localized to the membrane is increased by RAB33A overexpression in cells.

The degradation process is a fundamental feature of the autophagic pathway and involves both bulk and selective degradation of cellular components. Degradative autophagy is essential for maintaining protein homeostasis and the physiological function of cells, and it plays a crucial role in the regulation of energy metabolism, the development of metabolic tissues, and the pathogenesis of metabolic disorders such as obesity and diabetes [34]. The induction of autophagy can eliminate damaged mitochondria, thereby inhibiting the generation of tumor cells [35]. In contrast, nondegradative autophagy, as exemplified by the ATG5-ATG12/ATG16L1 protein complex, plays a pivotal role in the antiviral effect of interferon gamma against murine norovirus. Viruses and bacteria adeptly evade degradation and induce nondegradative autophagy while simultaneously harnessing autophagic vacuoles to support their survival, replication, and dissemination. For example, Kaposi’s sarcoma-associated herpesvirus interacts with Rubicon and inhibits the fusion of the autophagosome with lysosome to enhance viral replication [36]. Similarly, Poliovirus induces nondegradative autophagy to form double-membrane vesicles to promote its replication. Importantly, the impairment of genes central to autophagosome formation differs from the loss of function of genes involved in autophagosome maturation, with the latter leading to milder defects and more gradual accumulation of autophagic vacuoles [17]. Additional pharmacological agents can induce nondegradative autophagy. AMDE-1 has emerged as a bifunctional compound that plays dual roles in both the activation and inhibition of autophagy [23]. Furthermore, the inhibition of lysosomal function triggers autophagy through a feedback mechanism that results in the downregulation of mTOR complex 1 (mTORC1) activity [37]. Notably, RAB33A can not only induce autophagy but also concurrently inhibit the degradation process of autophagy, which is similar to the dual roles of AMDE-1 in autophagy. In addition, secretory autophagy represents another nondegradative function of the autophagy machinery.

## Supplementary information

supplementary information

original western blots

## Data Availability

The data generated in this work are available within the article and its supplementary data files. The authenticity of this manuscript was validated by uploading the key raw data to the Research Data Deposit public platform (www.researchdata.org.cn). Any additional information related to this study is available from the corresponding author on reasonable request.
