# Supplementary material for: RAB33A promotes metastasis via RhoC accumulation through non-canonical autophagy in cervical cancer
Source: Cell Death Dis. 2025 Feb 25;16(1):130. doi: 10.1038/s41419-025-07455-w (PMC11861591; doi:10.1038/s41419-025-07455-w)
Supplement: Supplementary file 1 — supplementary information [file 41419_2025_7455_MOESM1_ESM.docx]

**Supplementary Figure and Figure legends**

**
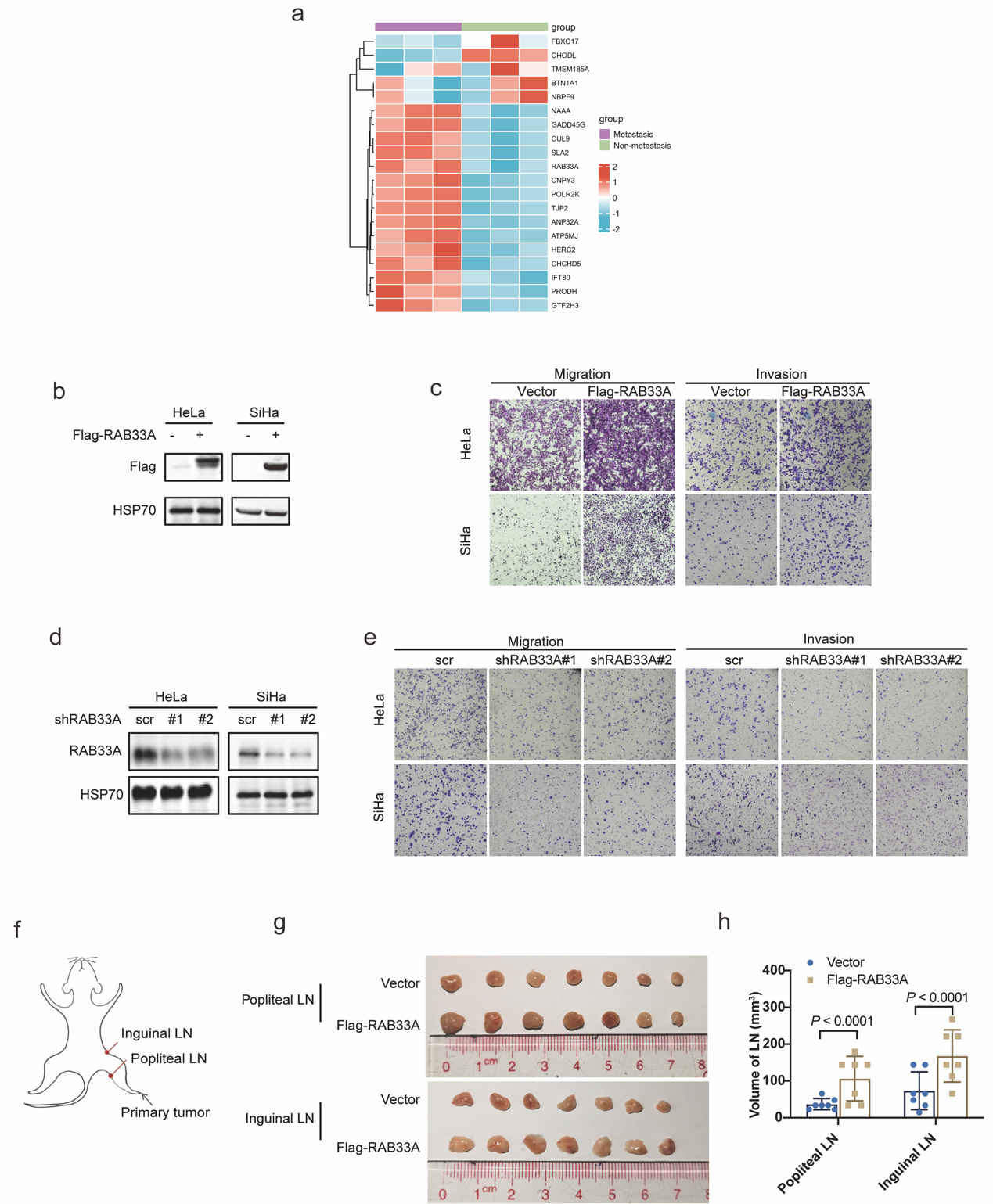
**

**Figure S1.** RAB33A promotes cervical cancer metastasis in vivo and in vitro.

**(a)** Representative heatmaps from global comparative transcriptome analysis in 3 cervical cancer tissues without metastasis (green) and 3 cervical cancer tissues with metastasis (purple).

**(b)** Western blotting analysis of RAB33A overexpressing HeLa or SiHa cells and wild-type.

**(c)** Migration and invasion of HeLa or SiHa cells overexpressing RAB33A.

**(d)** Western blotting analysis of RAB33A-knockdown HeLa or SiHa cells and wild-type.

**(e)** Migration and invasion of HeLa or SiHa cells with or without RAB33A knockdown.

**(f)** Image of a nude mouse model with plantar popliteal inguinal lymph node (LN) metastasis.

**(g and h)** In vivo model of cervical cancer lymph node metastasis established using the indicated stable cell lines; the model was established in 7 biologically independent mice. Dissected popliteal and inguinal LNs **(g)** and their volumes **(h)** from mice with tumors derived from wild-type or RAB33A-overexpressing HeLa cells.


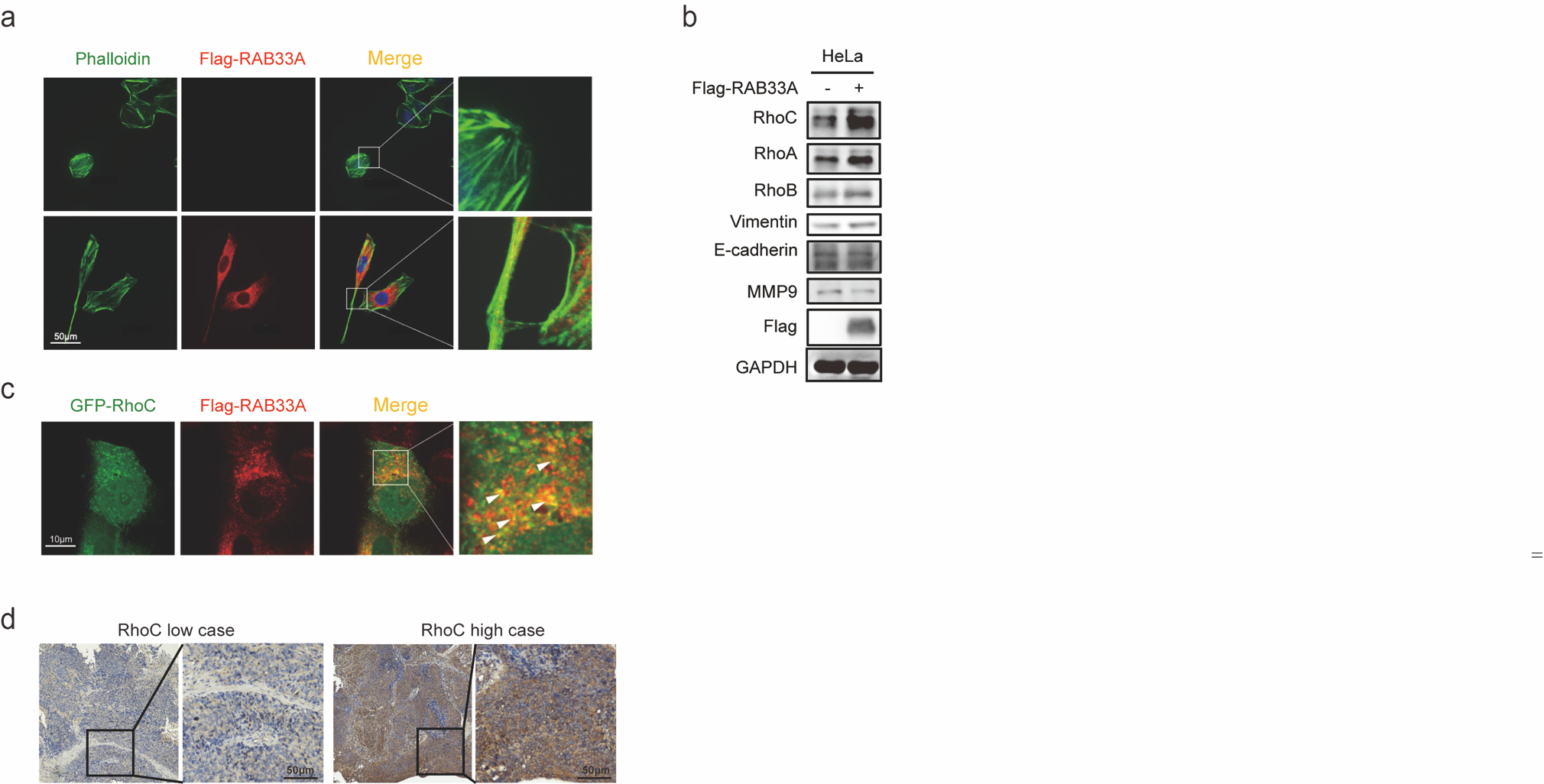


**Figure S2.** RhoC is required for RAB33A-mediated cervical carcinoma metastasis.

**(a)** Immunofluorescence staining demonstrating cytoskeletal changes, as indicated by enhanced linear and patchy pseudopodia formation, upon RAB33A overexpression. Immunofluorescence analysis of HeLa cells overexpressing Flag-RAB33A. F-actin was stained with phalloidin (green).

**(b)** RAB33A overexpression increases RhoC expression, as shown by Western blotting analysis. The protein levels of RhoA, RhoB and RhoC in wild-type or Flag-RAB33A-overexpressing HeLa cells were measured via Western blotting.

**(c)** Immunofluorescence staining showing RhoC and RAB33A colocalization (white arrow). Localization of GFP-RhoC and Flag-RAB33A (red) in HeLa cells. Arrowheads indicate the colocalization of the two proteins.

**(d)** Immunohistochemical staining for RhoC in cervical carcinoma specimens delineating cases without metastasis or recurrence from those with distant metastasis.

**
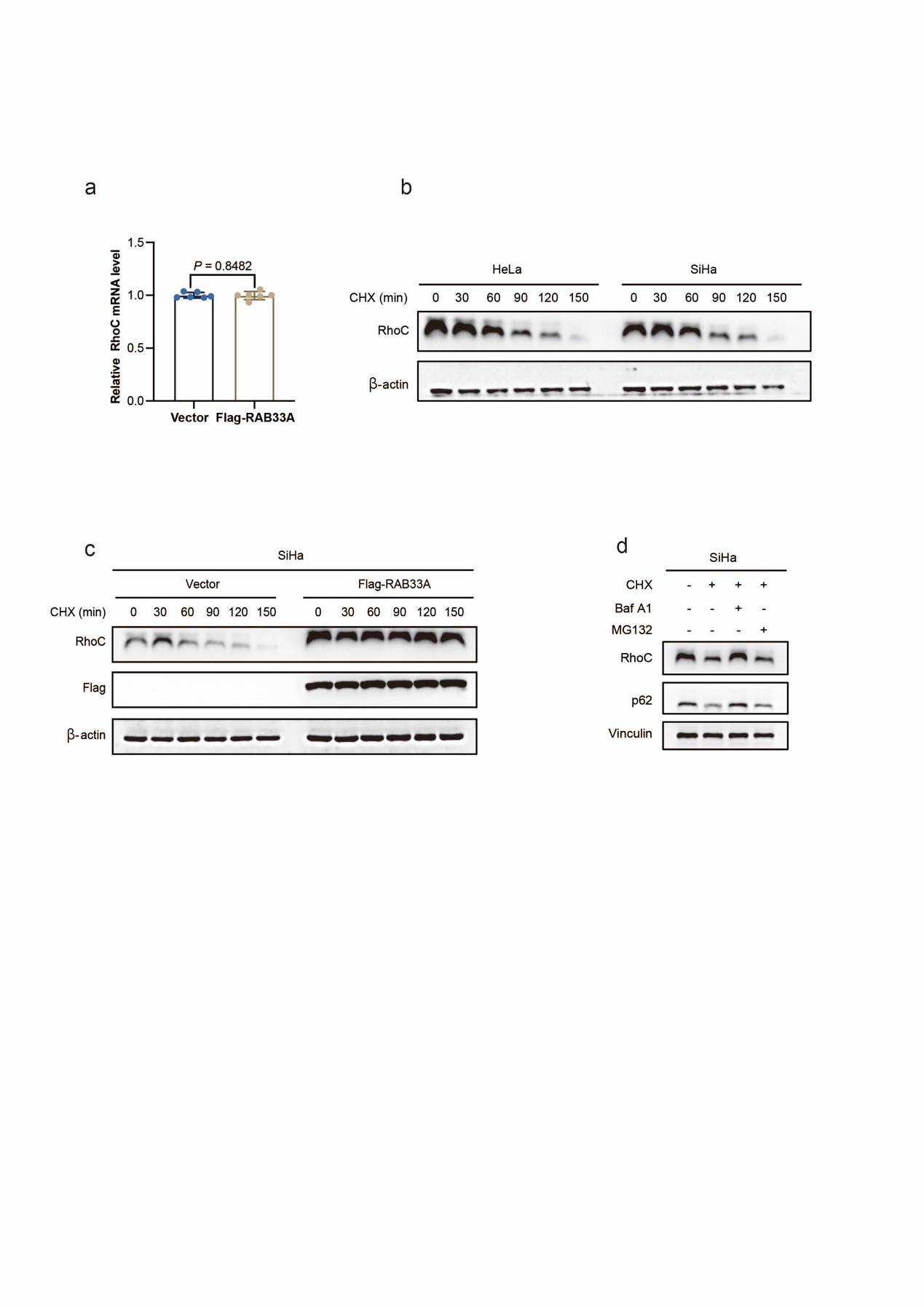
**

**Figure S3.** RAB33A stabilizes the protein level of RhoC via the autophagy pathway.

**(a)** qPCR showing no increase in the mRNA levels of RhoC in response to RAB33A overexpression.

**(b)** Western blotting analysis of the RhoC protein levels in wild-type or RAB33A-overexpressing SiHa cells treated with 40 μg/mL CHX.

**(c)** RhoC undergoes autophagy-mediated degradation (CHX: 40 μg/mL, 6 hours; Baf A1: 100 nM, 6 hours; MG132: 10 μM, 6 hours). SiHa cells were treated with the indicated inhibitors for 6 hours, after which the RhoC protein levels were determined via Western blotting.

**(d)** The half-life of RhoC was 90 minutes, as determined by Western blotting. Changes in the RhoC protein levels in HeLa and SiHa cells upon treatment with CHX for the indicated times.

**
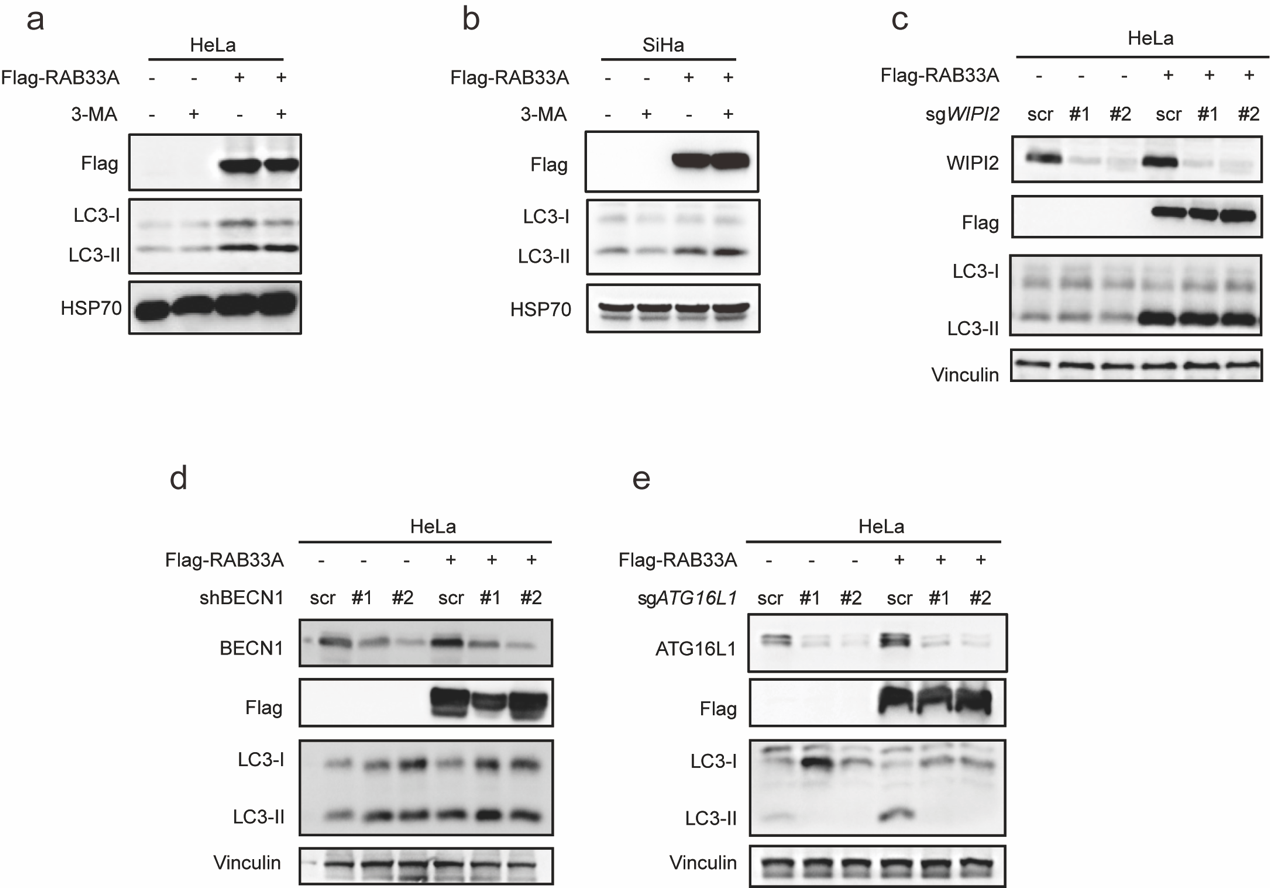
**

**Figure S4.** RhoC is degraded via the autophagy-lysosomal pathway after interacting with LC3 via LIR motifs.

**(a and b)** LC3-II upregulation was not inhibited by 3-MA (10 mM, 6 hours). Wild-type or RAB33A-overexpressing HeLa **(a)** and SiHa **(b)** cell lines were treated with the PI3K inhibitor 3-MA for 6 hours. The protein levels of LC3-II were determined by Western blotting.

**(c and d)** Western blots of Flag-RAB33A HeLa cell lysates showing the effects of *WIPI2* knockout **(c)** and *BECN1* knockout **(d)** using sgRNAs.

**(e)** Western blotting analysis of RhoC and LC3-II levels in HeLa cells with *ATG16L1* knockout combined with RAB33A overexpression.

**
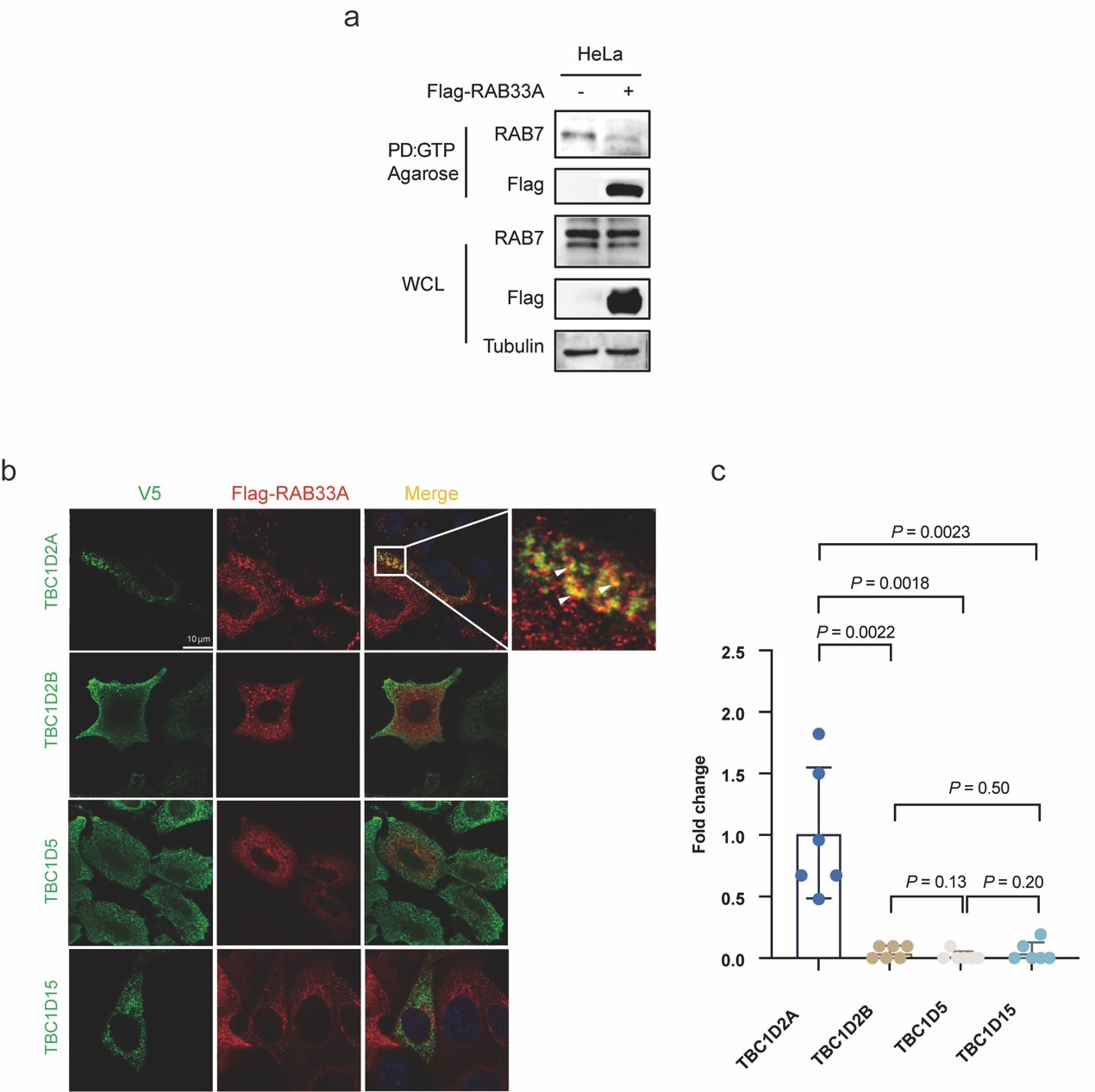
**

**Figure S5.** TBC1D2A is recruited by RAB33A to deactivate RAB7 and prevent RhoC degradation.

**(a)** Active RAB7 in wild-type or RAB33A-overexpressing HeLa cells was detected by a GTP pull-down assay.

**(b)** Immunofluorescence analysis of the colocalization of RAB33A and V5-TBC1D2A, V5-TBC1D2B, V5-TBC1D5 or V5-TBC1D15 in HeLa cells stably overexpressing RAB33A. Arrowheads indicate the colocalization of two proteins. The cells were transiently transfected with the indicated plasmids for 48 hours.

**(c)** The colocalization of RAB33A with V5-TBC1D2A, V5-TBC1D2B, V5-TBC1D5 or V5-TBC1D15 was quantified (*n*= 6 fields). The data are presented as the mean ± SD. *P* values are shown; Student’s *t*-test.

Supplementary Table 1：sgRNA sequences for CRISPR knock out and shRNA sequences for knock down.

| **Genes** | **Type** | **Sequence** |
| --- | --- | --- |
| ATG5 | sgRNA | GATGTAGTGTTGGAACATGT |
| ATG7 | sgRNA | ACAAGCCCAAGAGAGGTTGG |
| ATG16L1 | sgRNA | CACACACTCACGGGACACAG |
| BECN1 | sgRNA | CCAAGTCCGGTCTACCGCGG |
| WIPI2 | sgRNA | GAGGCTGATAGTATGCCTGG |
| RAB33A | sgRNA | ATCTCCCCCGGACCGAACCGGGG |
|  | shRNA1 | GACATCTTTCACCAACCTCAA |
|  | shRNA2 | GCCCACAACATGCTCTTGTTT |
| RhoC | sgRNA | AGATCGGGGCCCGTGCGAGGAGG |
