# Supplementary material for: RAB33A promotes metastasis via RhoC accumulation through non-canonical autophagy in cervical cancer
Source: Cell Death Dis. 2025 Feb 25;16(1):130. doi: 10.1038/s41419-025-07455-w (PMC11861591; doi:10.1038/s41419-025-07455-w)

Supplemental Material- Original Western Blots

Relevant areas for cropped blots in the main and Supplementary figures are shown with a dashed box.

Figure 2a

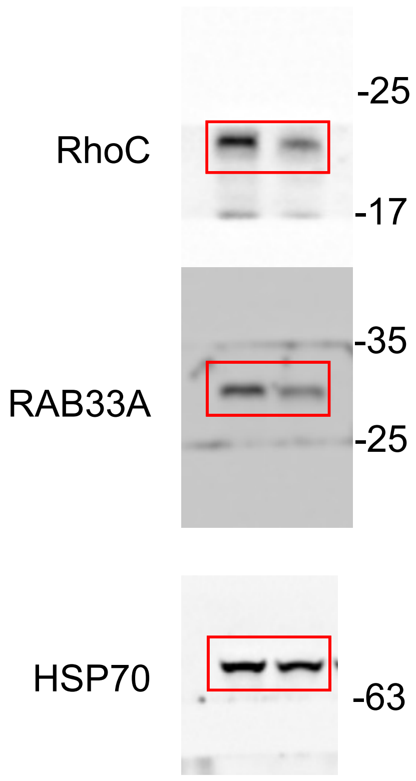

Figure 2c

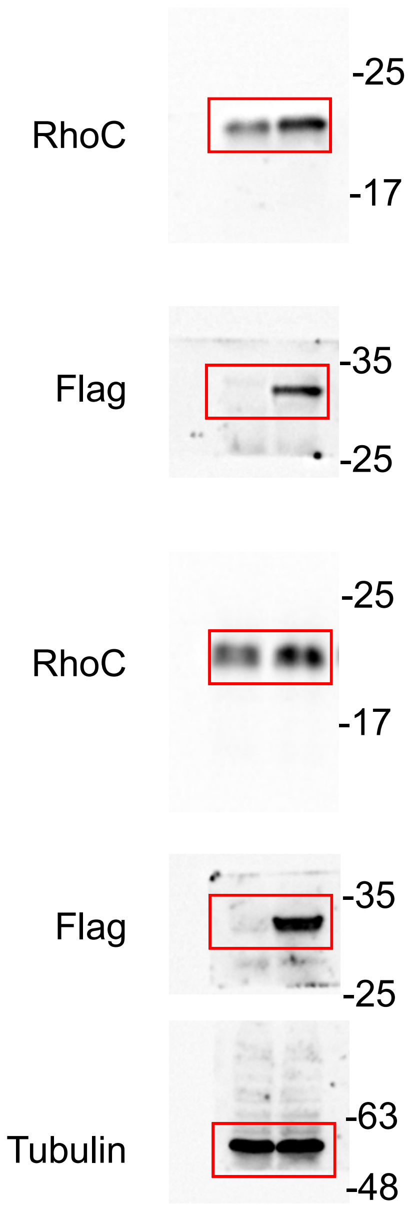

Figure 2e

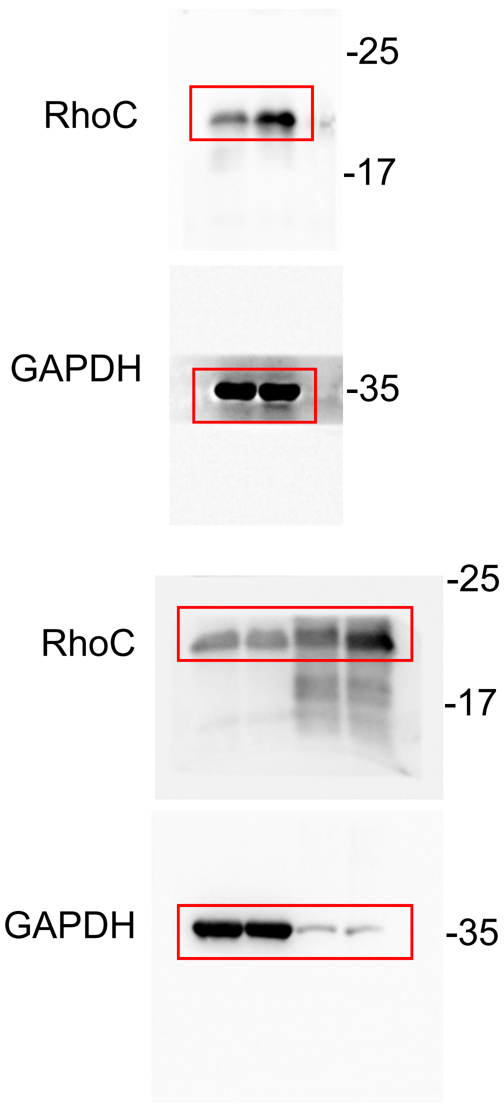

Figure 2g

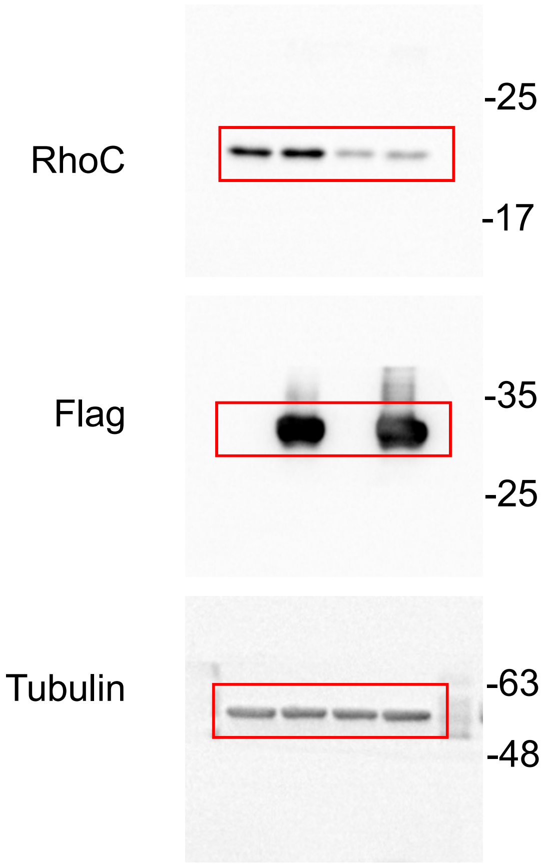

Figure 3a

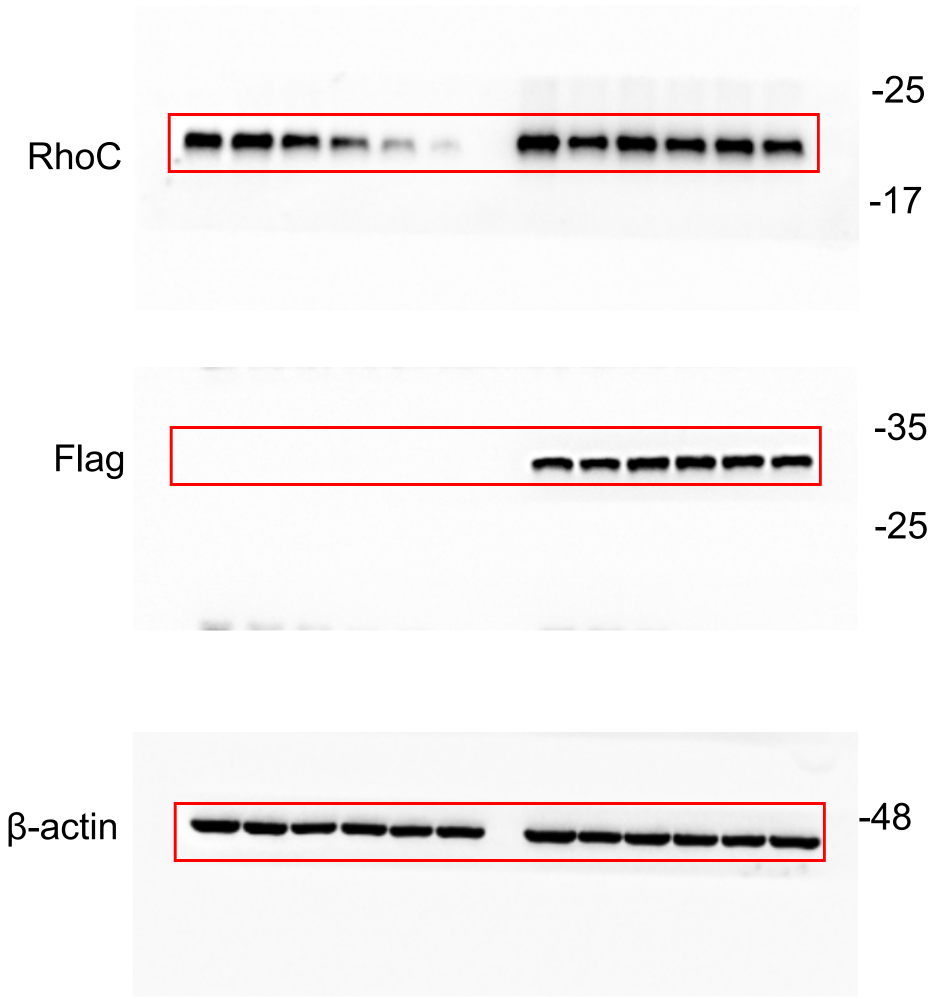

Figure 3c

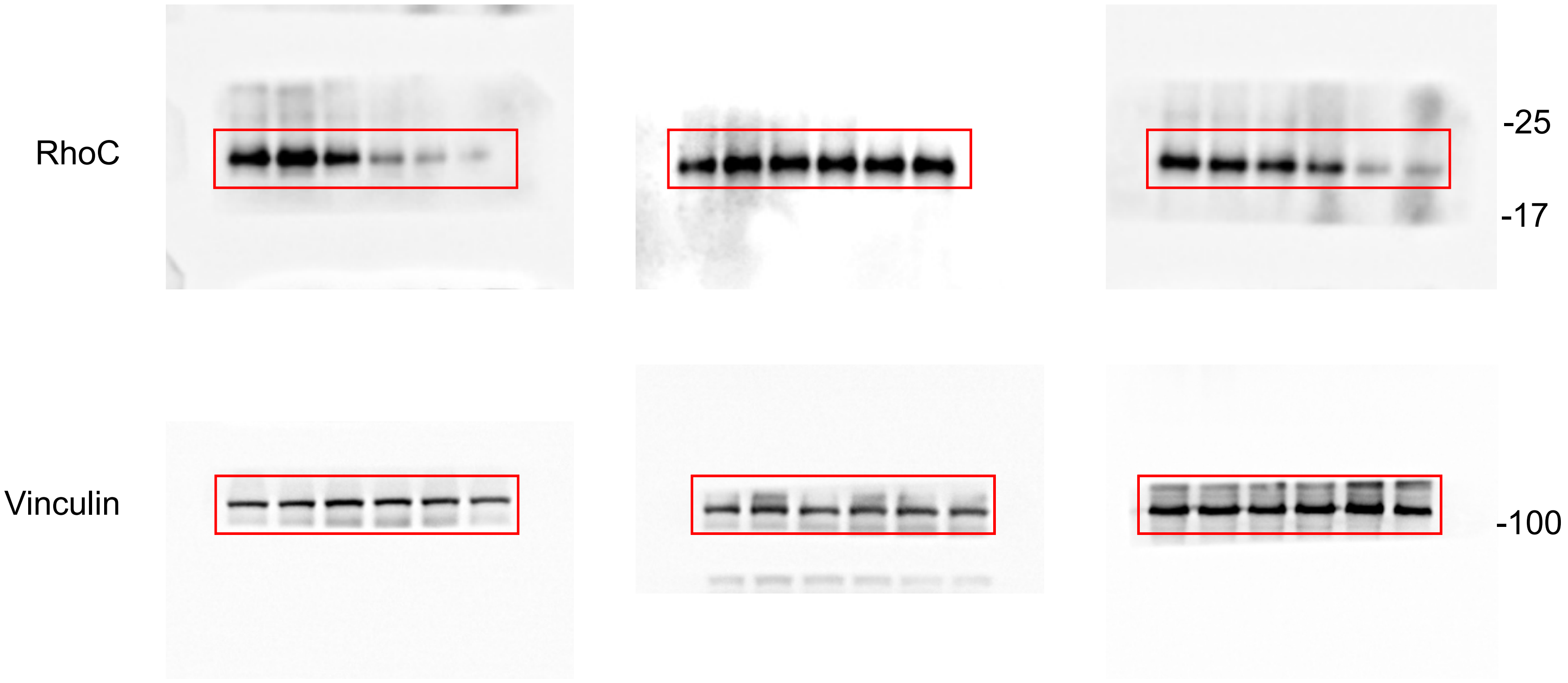

Figure 3e

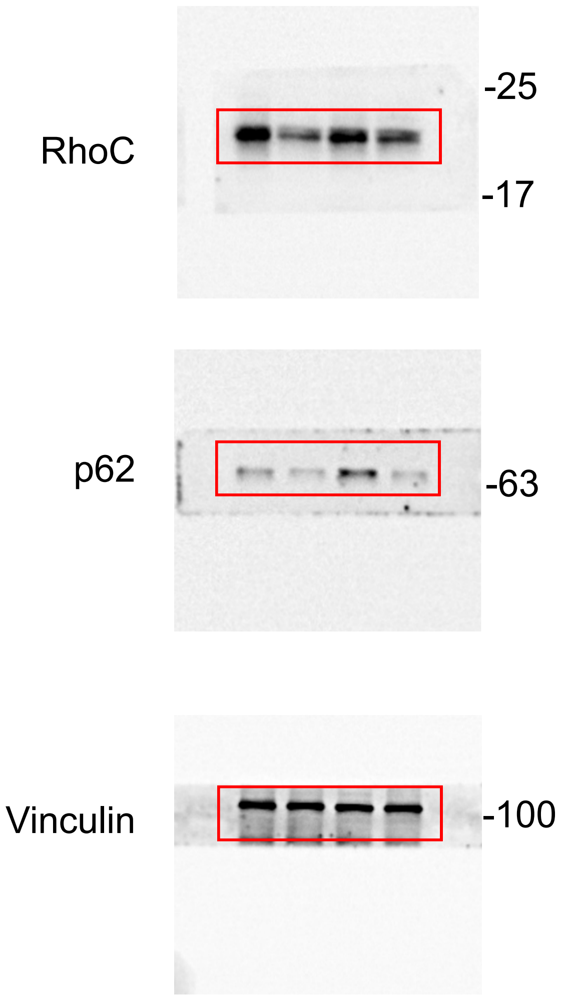

Figure 3i

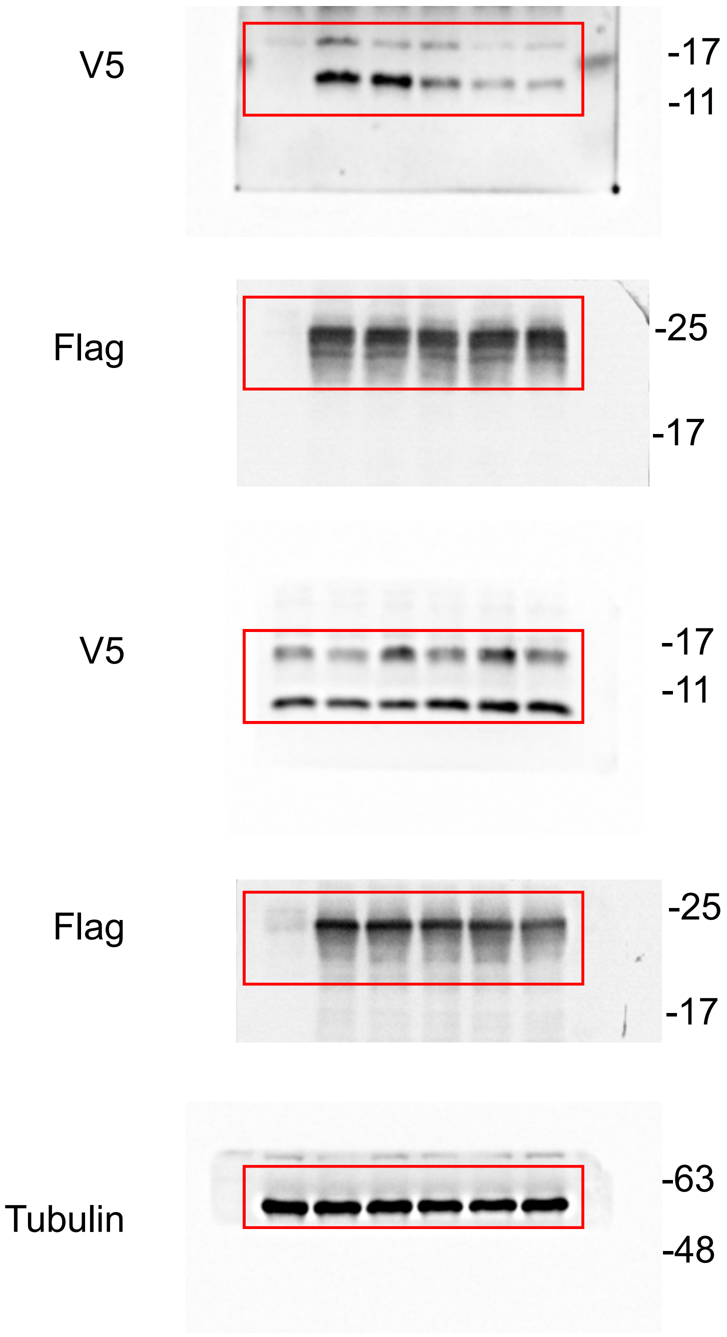

Figure 4a

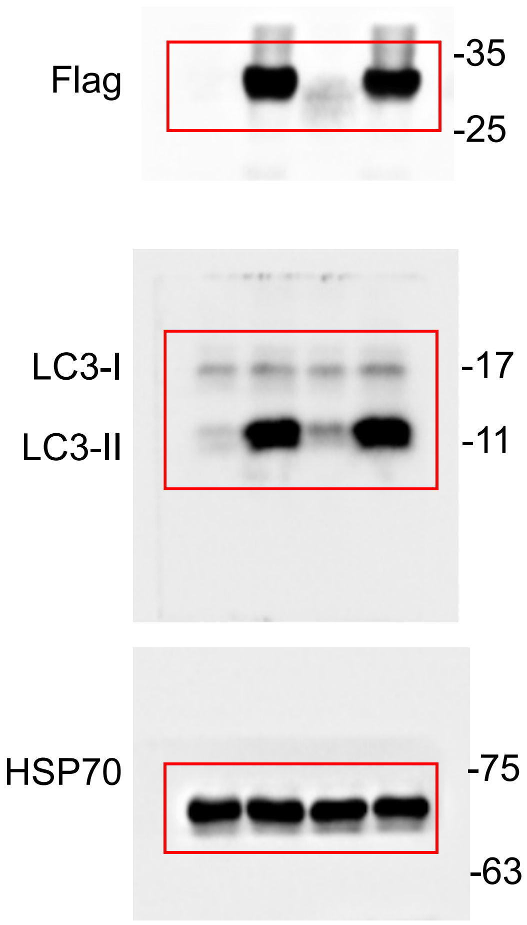

Figure 4c

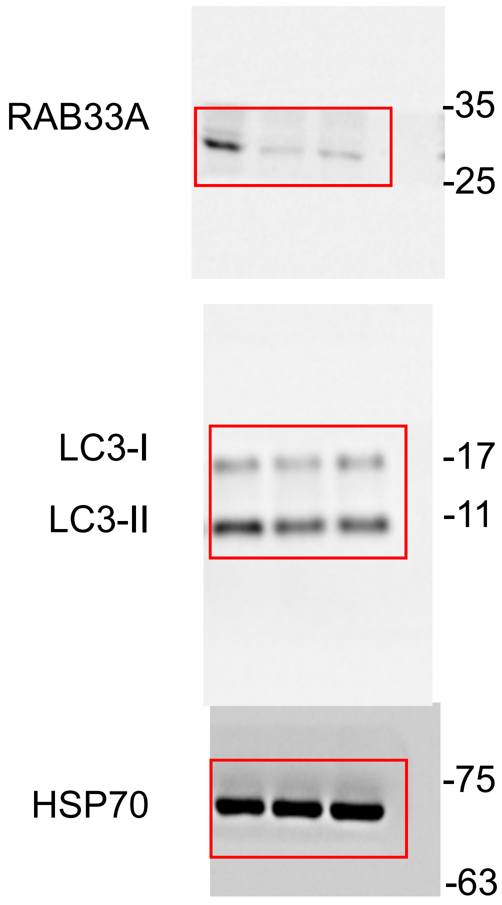

Figure 4e

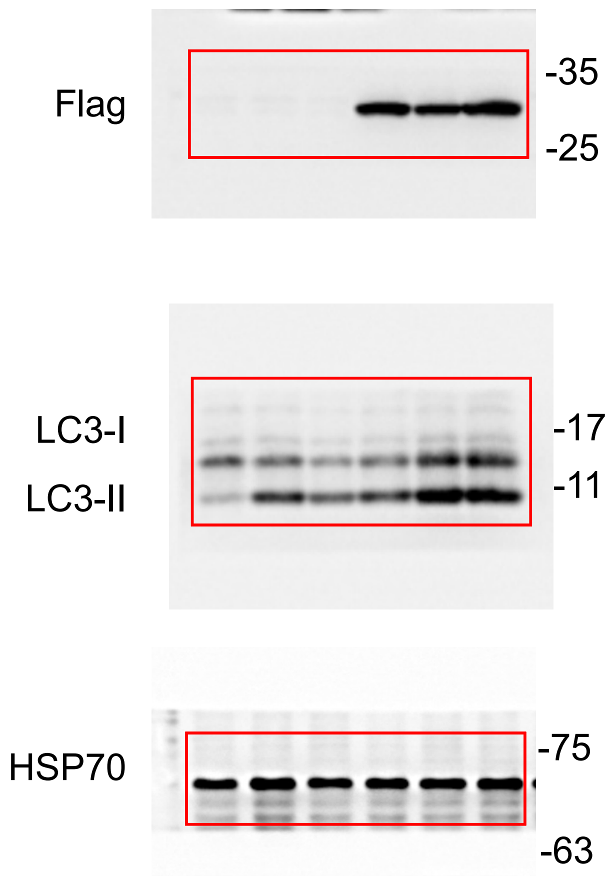

Figure 4g

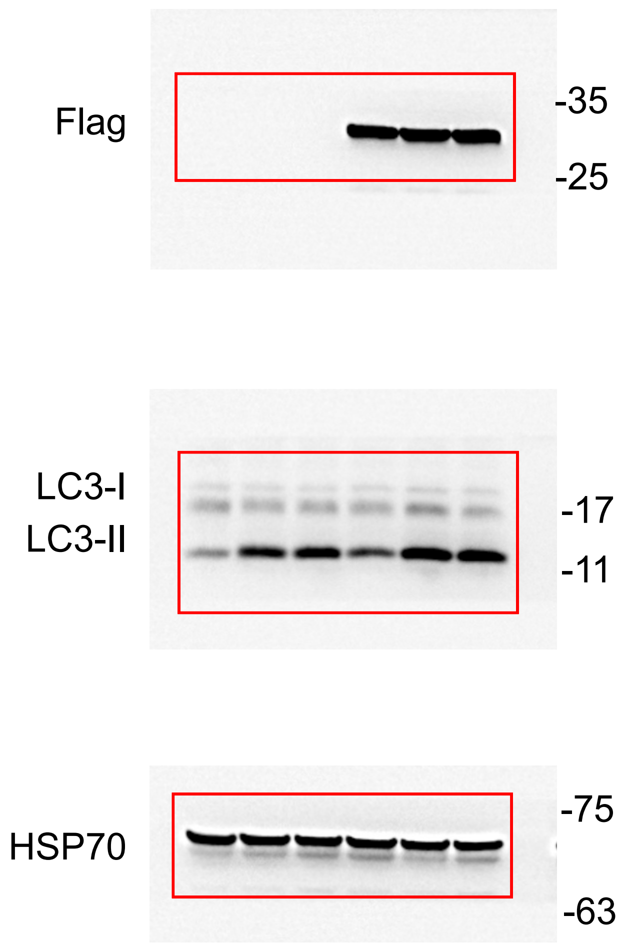

Figure 4n

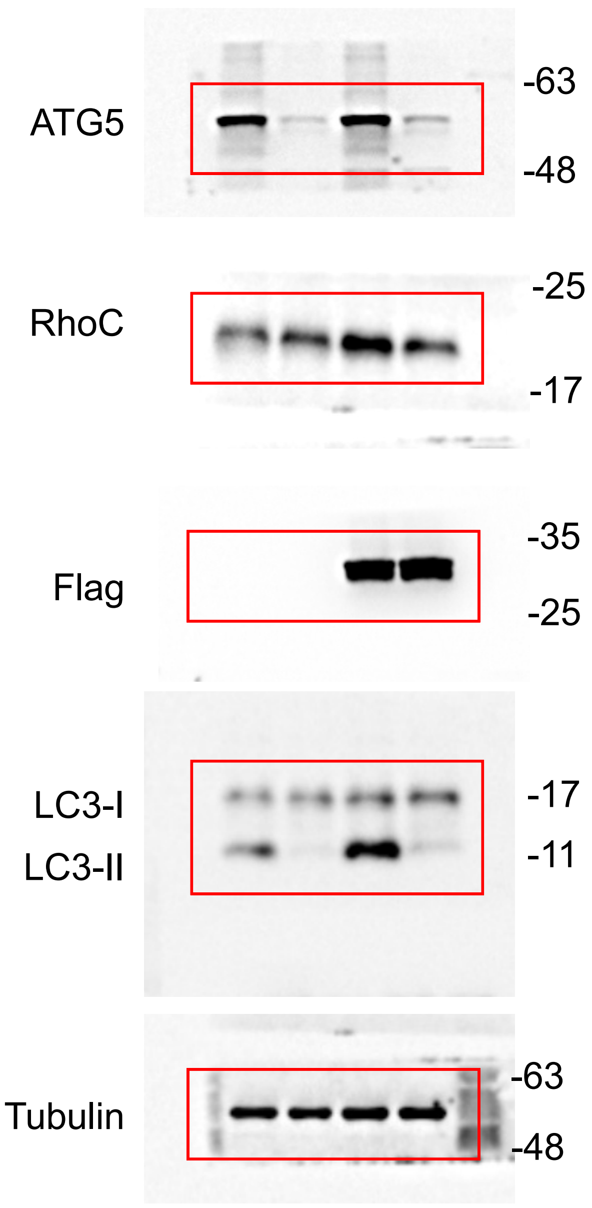

Figure 4q

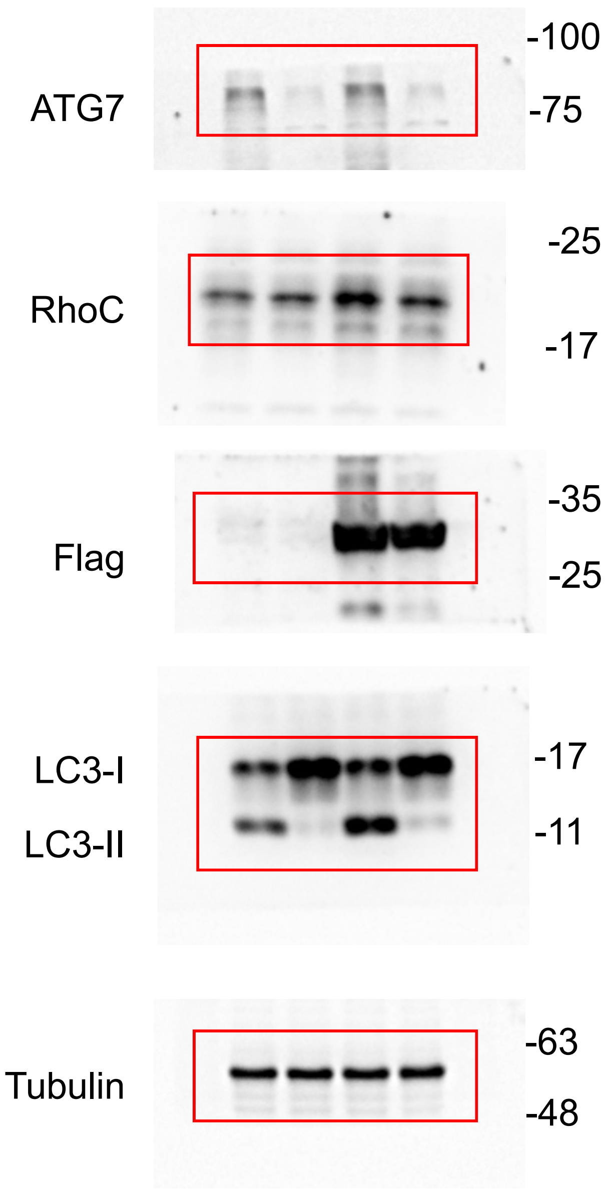

Figure 5c

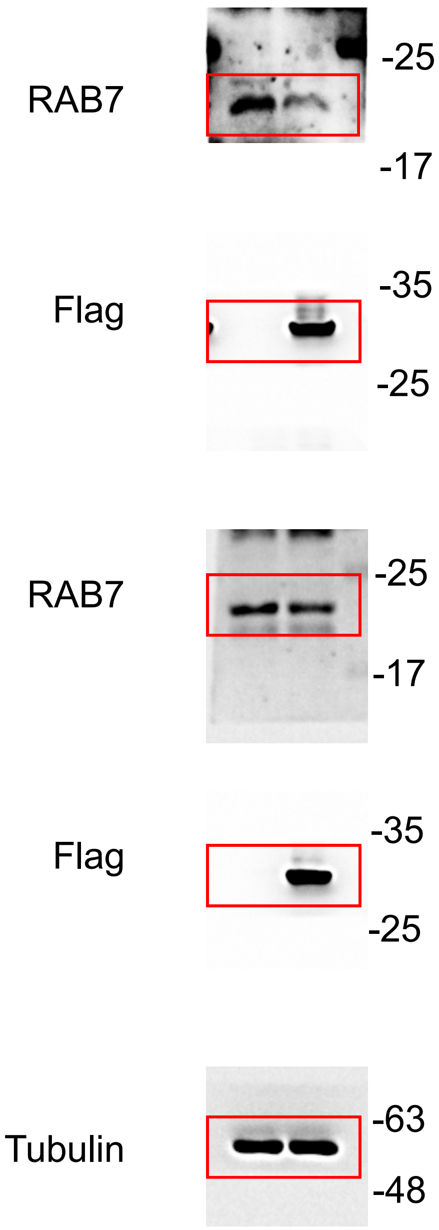

Figure 5f

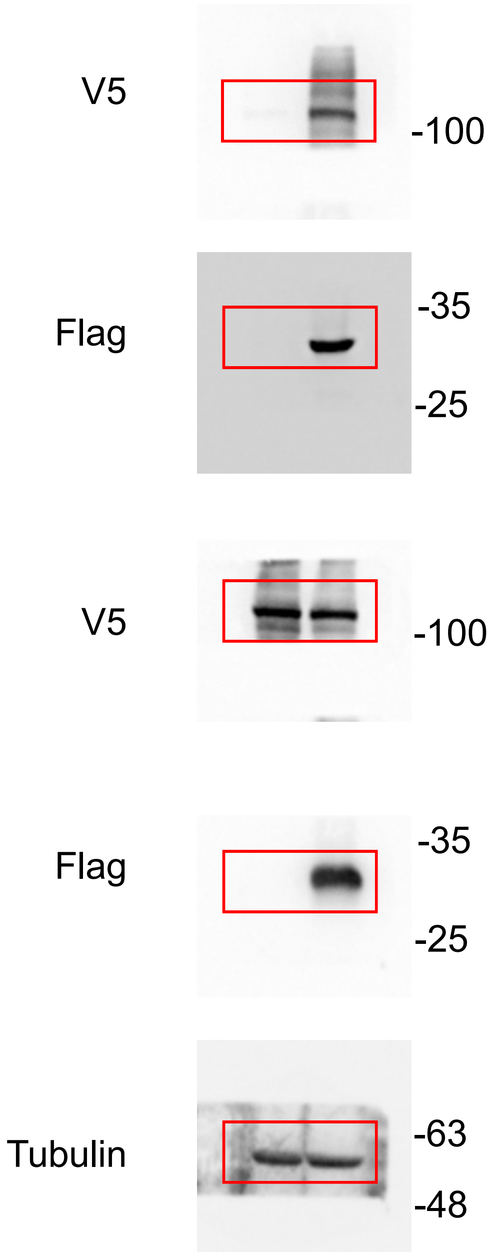

Figure 5g

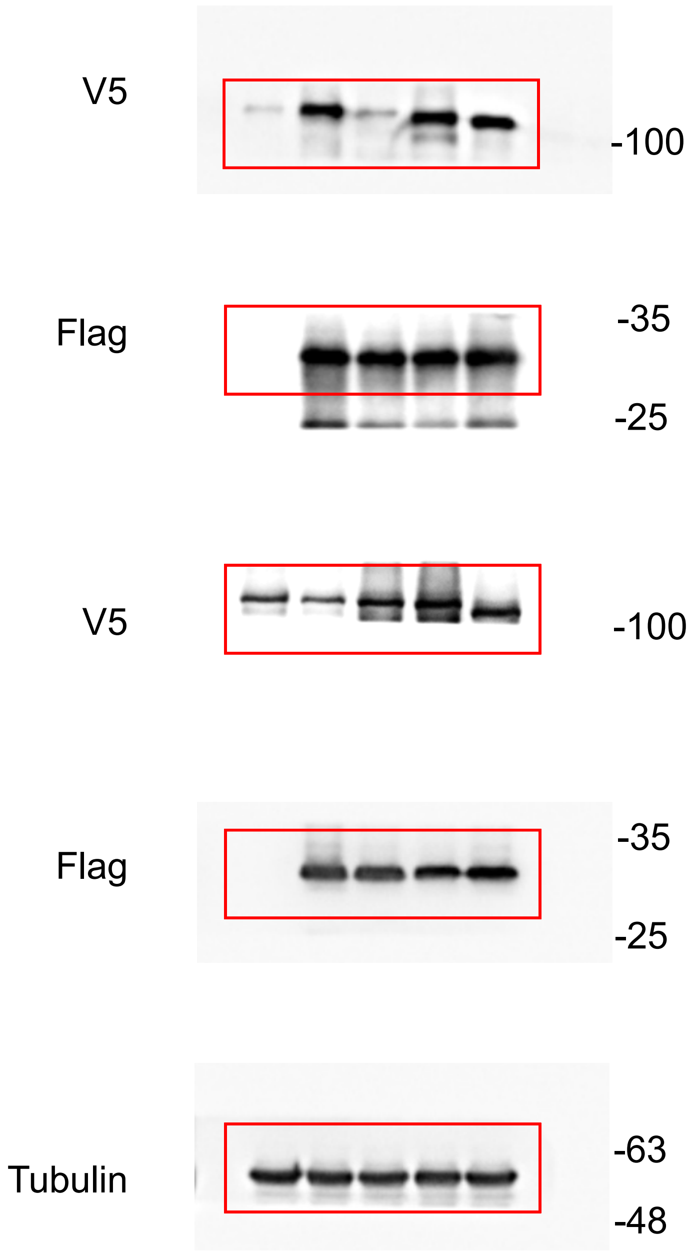

Figure S1b

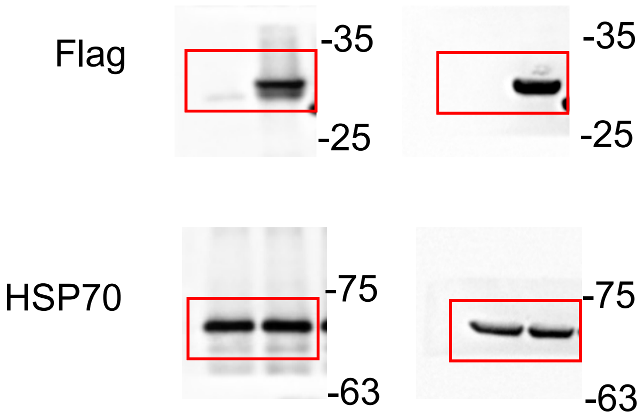

Figure S1d

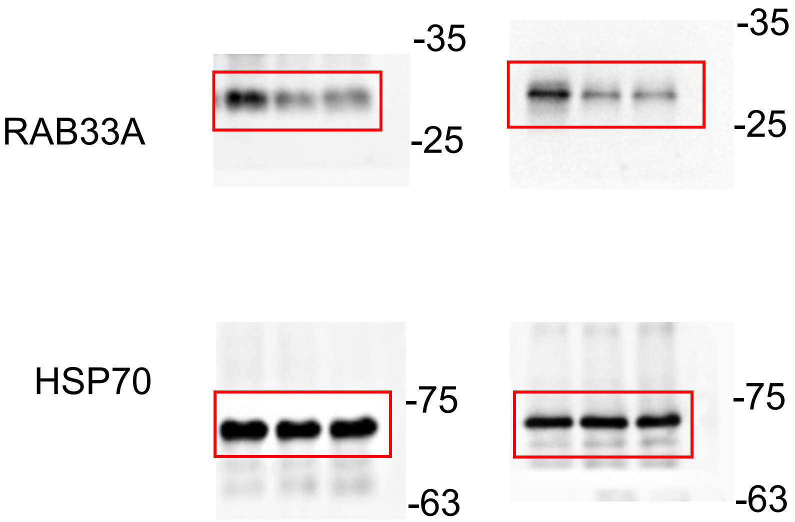

Figure S2b

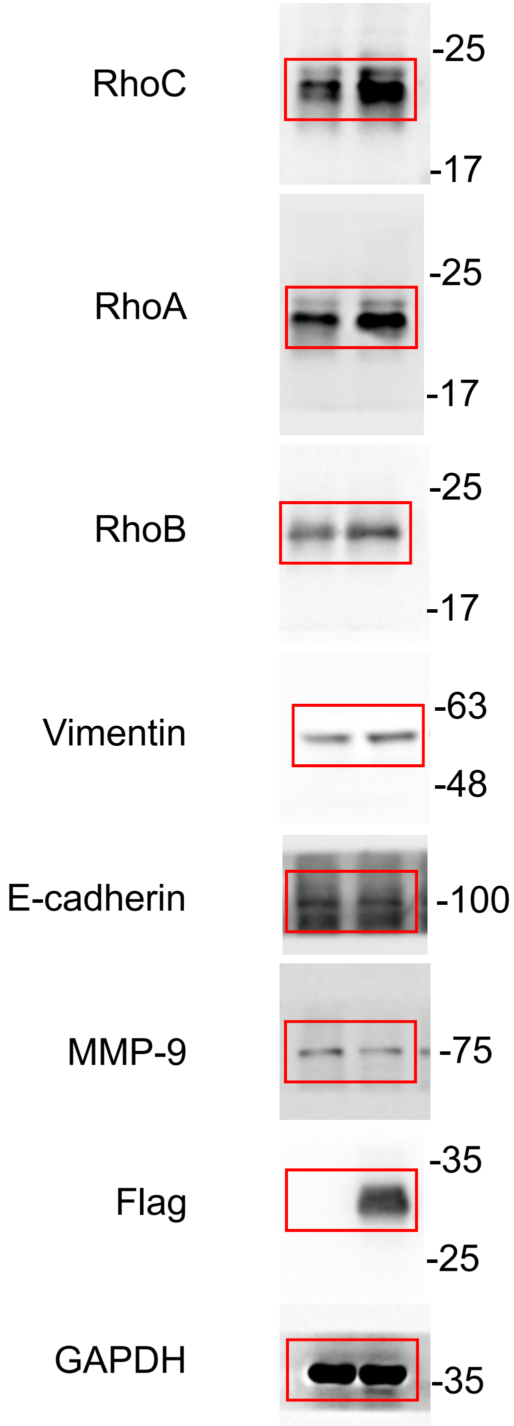
RhoC
-25
-17
RhoA
-25
-17
RhoB
-25
-17
Vimentin
-63
-48
E-cadherin
-100
MMP-9
-75
Flag
-35
-25
GAPDH
-35

Figure S3b

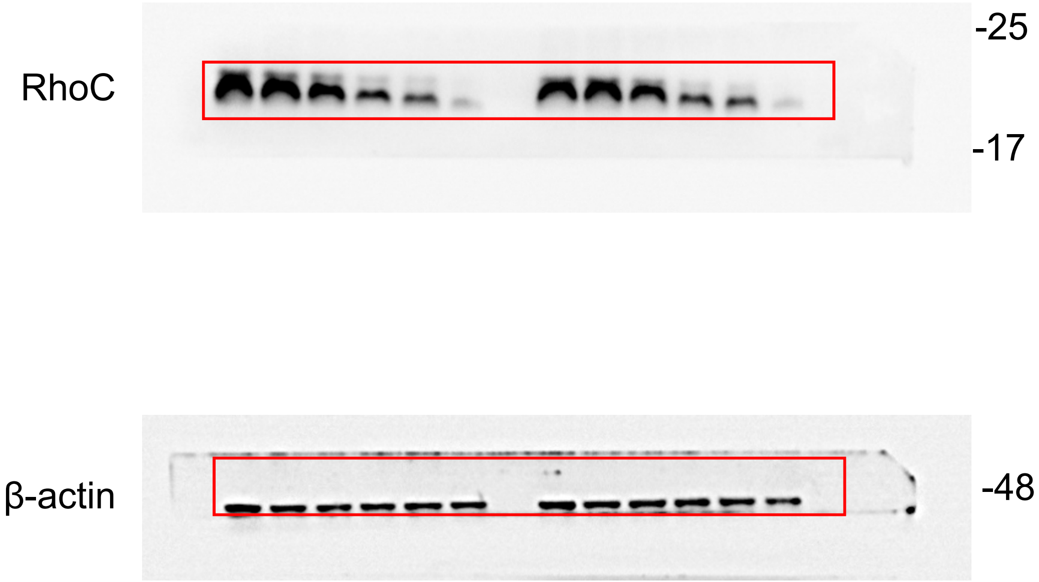

Figure S3c

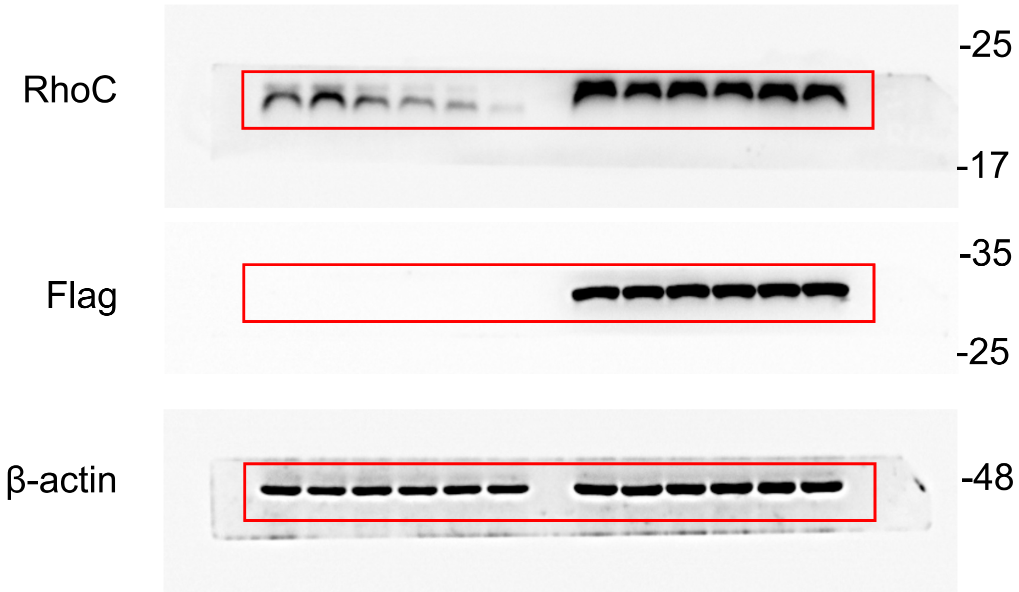

Figure S3d

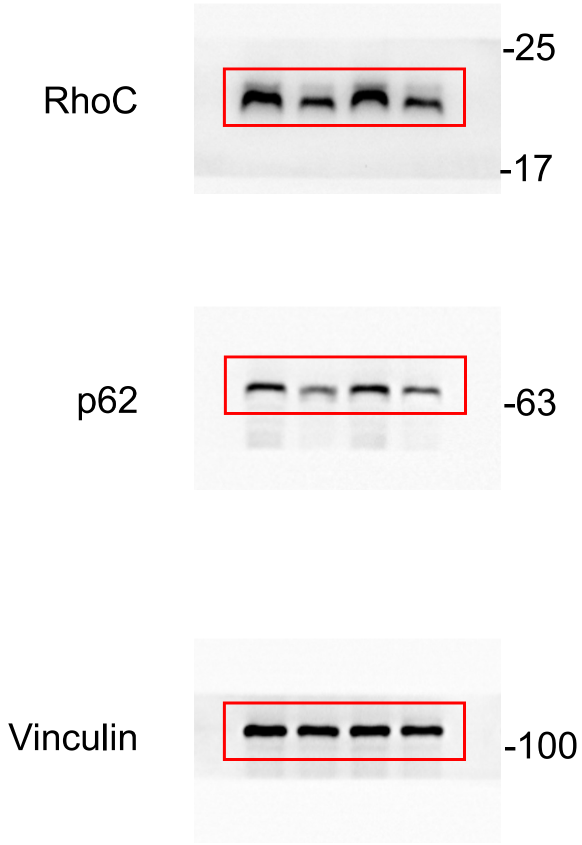

Figure S4a

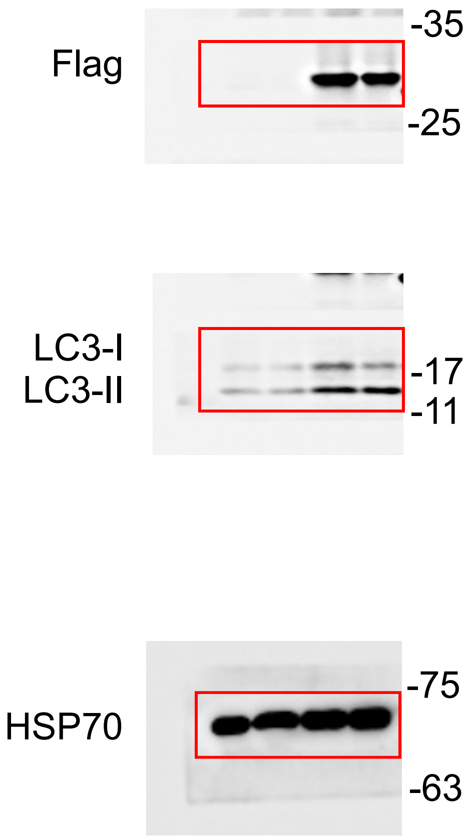

Figure S4b

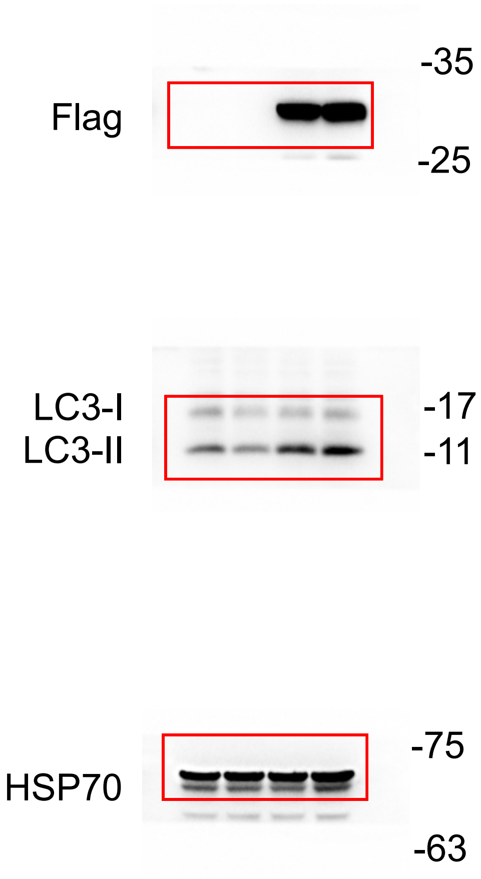

Figure S4c

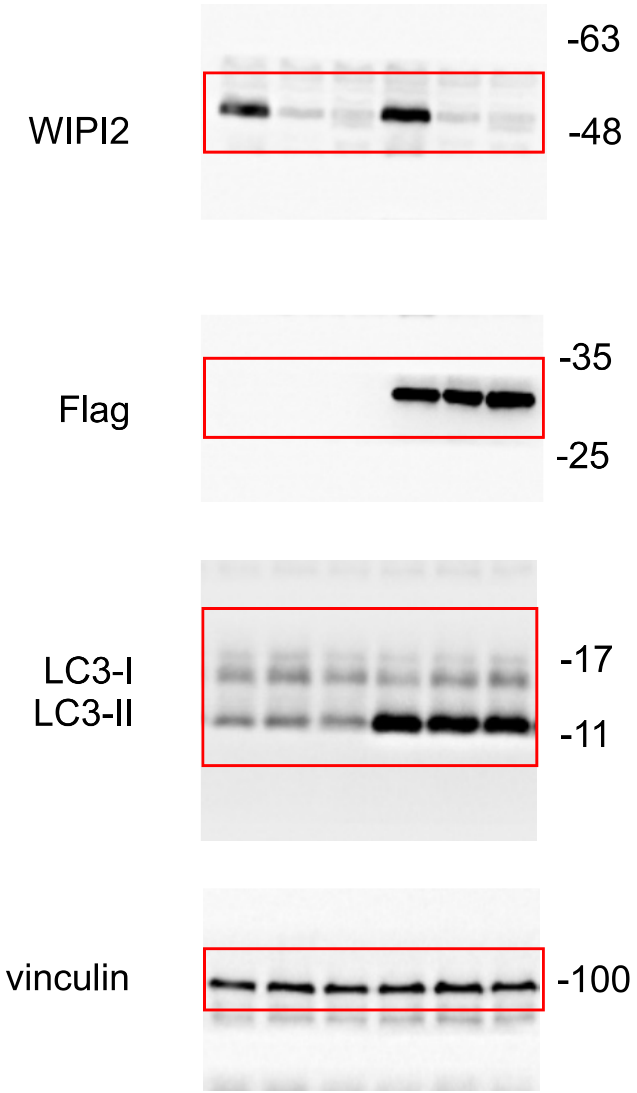

Figure S4d

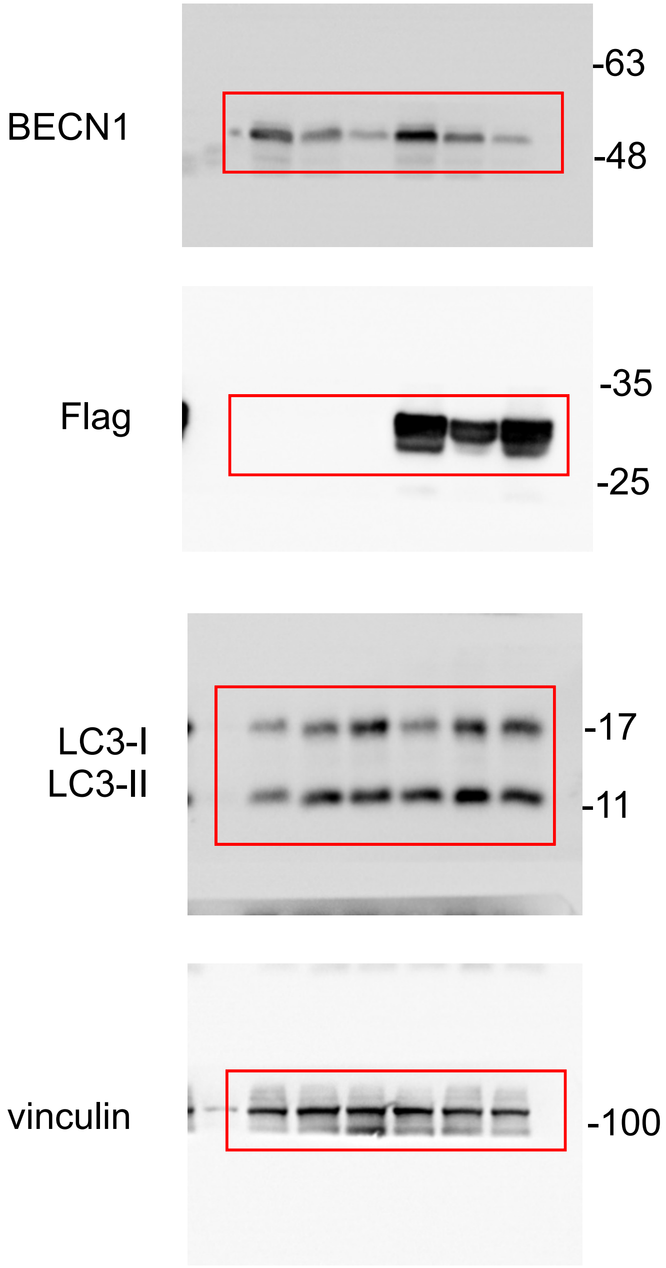

Figure S4e

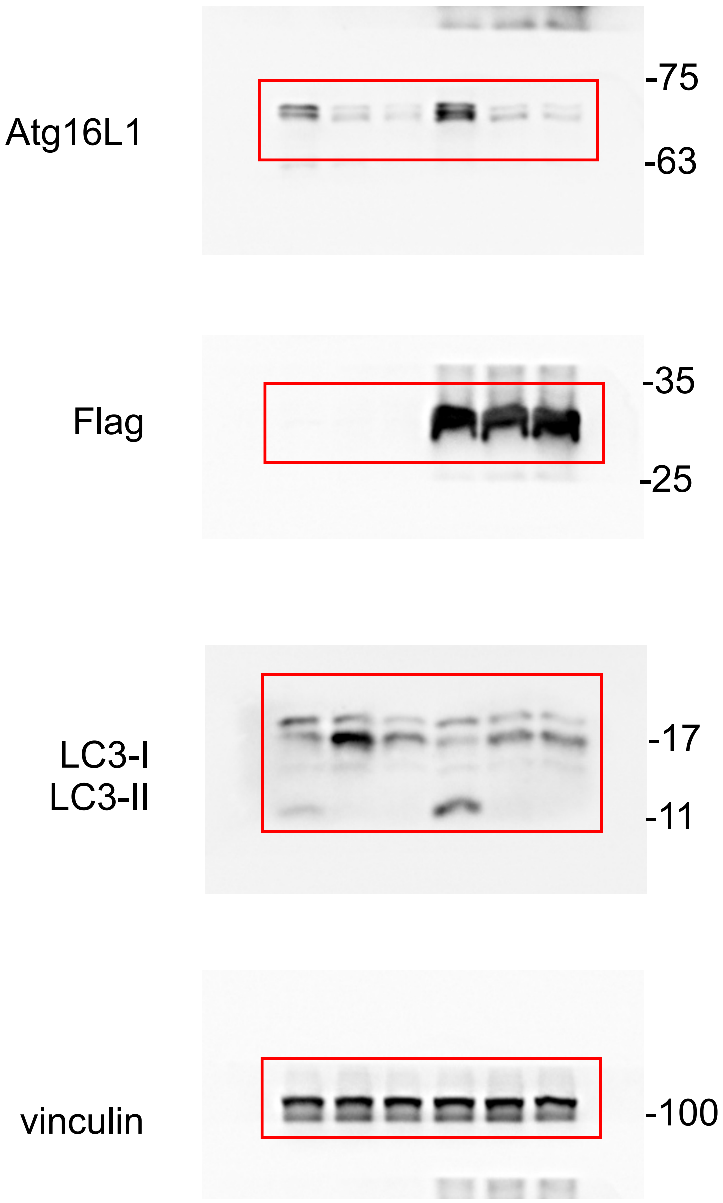

Figure S5a

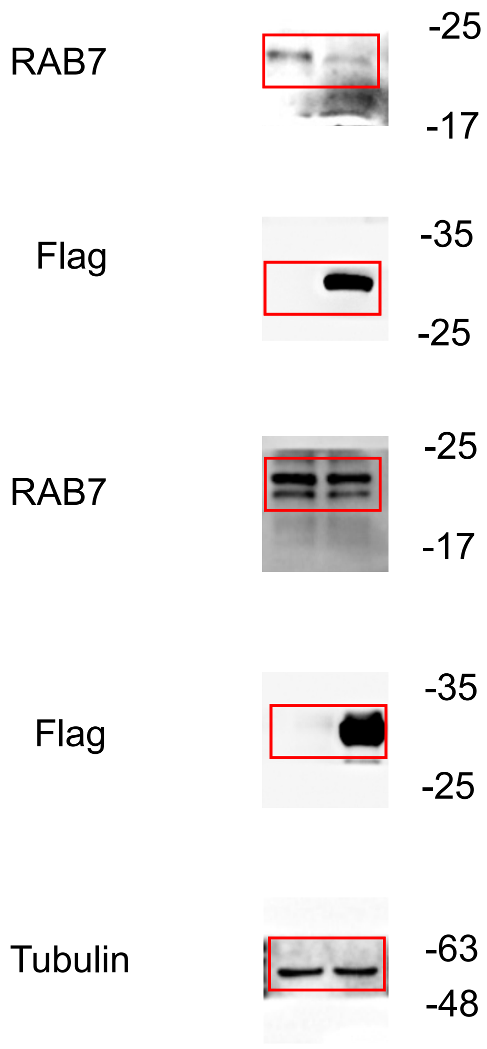

Supplement: Supplementary file 2 — original western blots [file 41419_2025_7455_MOESM2_ESM.pdf]
